# The role of apelinergic system in metabolism and reproductive system in normal and pathological conditions: an overview

**DOI:** 10.3389/fendo.2023.1193150

**Published:** 2023-06-22

**Authors:** Keyvan Mehri, Gholamreza Hamidian, Zohreh Zavvari Oskuye, Sepehr Nayebirad, Fereshteh Farajdokht

**Affiliations:** ^1^ Drug Applied Research Center, Tabriz University of Medical Sciences, Tabriz, Iran; ^2^ Department of Basic Sciences, Faculty of Veterinary Medicine, University of Tabriz, Tabriz, Iran; ^3^ Tehran Heart Center, Cardiovascular Diseases Research Institute, Tehran University of Medical Sciences, Tehran, Iran; ^4^ Neurosciences Research Center, Tabriz University of Medical Sciences, Tabriz, Iran

**Keywords:** apelin, obesity, reproduction, diabetes, insulin sensitivity

## Abstract

Lifestyle changes have made metabolic disorders as one of the major threats to life. Growing evidence demonstrates that obesity and diabetes disrupt the reproductive system by affecting the gonads and the hypothalamus-pituitary-gonadal (HPG) axis. Apelin, an adipocytokine, and its receptor (APJ) are broadly expressed in the hypothalamus nuclei, such as paraventricular and supraoptic, where gonadotropin-releasing hormone (GnRH) is released, and all three lobes of the pituitary, indicating that apelin is involved in the control of reproductive function. Moreover, apelin affects food intake, insulin sensitivity, fluid homeostasis, and glucose and lipid metabolisms. This review outlined the physiological effects of the apelinergic system, the relationship between apelin and metabolic disorders such as diabetes and obesity, as well as the effect of apelin on the reproductive system in both gender. The apelin–APJ system can be considered a potential therapeutic target in the management of obesity-associated metabolic dysfunction and reproductive disorders.

## 1 Introduction

Sedentary lifestyles and dietary changes in recent decades have increased the risk of metabolic disorders such as obesity and diabetes worldwide (1). As a result, more than a third of the world’s population is overweight and obese (2).

Obesity is a common metabolic disorder, which is defined as an abnormally high or excessive accumulation of fat and hence a high body mass index (BMI) (3, 4). The World Health Organization (WHO) defines BMI of ≥ 25 kg/m2 as overweight, ≥ 30 kg/m2 as obese, and ≥ 40 kg/m2 as severely obese (3). A Western diet and reduced physical activity are the primary environmental factors that cause obesity, in addition to genetics (5).

Obesity caused by a high-fat diet (HFD) can increase the risk of cardiovascular diseases and metabolic disorders such as type 2 diabetes mellitus (T2DM) and dyslipidemia (4). Many studies have shown that obesity or diabetes in both genders is associated with reproductive dysfunction and infertility by disrupting the hypothalamic-pituitary-gonadal (HPG) axis, as well as by reducing the number and quality of sperm and oocytes (3).

Adipose tissue, the main site of lipid storage, is involved in the production of adipokines or adipocytokines, such as leptin, adiponectin, resistin, visfatin, and apelin, which play a critical role in the control of glucose and lipid metabolism, insulin sensitivity, energy metabolism, immune system, and neuroendocrine function (6–9).

Apelin is a ligand of the G-protein-coupled receptor (GPCR) and regulates several biological functions in the human and rodents body, including blood pressure, fluid homeostasis, food intake and energy balance, immune response, and neuroendocrine response to stress *via* its autocrine, paracrine, endocrine, and exocrine effects (10, 11). Apelin is produced by adipose tissue, and many other tissues like the central nervous system, heart, kidneys, lungs, mammary glands, and placenta (12, 13). Moreover, its receptor (APJ) is broadly distributed in the lungs, cardiovascular system, kidney, mammary glands, white adipocytes, central nervous system, paraventricular nuclei, gastric mucosa, testes, and uterus (14, 15).

Of note, apelin plays an important regulatory role in glucose metabolism with its insulin-mimetic effects and lipid metabolism by promoting fuel consumption and reducing fat mass (16). According to previous studies, obesity and T2DM by inducing insulin resistance increase apelin levels (17). Moreover, previous studies indicated that the serum levels of apelin and its receptor gene expression can be influenced by diet (18, 19). Apelin expression is also influenced by nutritional status and its plasma levels decrease with fasting and increase with re-feeding (16).

The role of apelinergic system on the HPG axis has been proven, and apelin has been widely described as an influential factor in controlling reproduction in both genders (20). Due to the presence of apelin and its receptor in reproductive-related areas such as the hypothalamus and pituitary gland, its role in controlling the secretion of LH (luteinizing hormone) and FSH (follicle-stimulating hormone) is understandable (21). Furthermore, apelin is associated with reproductive disorders such as polycystic ovary syndrome (PCOS), endometriosis, and ovarian cancer (22).

This review outlines the current knowledge regarding the role of the apelinergic system in different physiological processes from the periphery to the brain, with an emphasis on the regulation of metabolism and metabolic disorders, namely diabetes and obesity. Moreover, its critical role in the physiology of the HPG axis and the reproductive system of both males and females was discussed.

## 2 Obesity and the reproductive system

Obesity can affect female reproductive processes, including ovarian follicular recruitment, oocyte growth and quality, oocyte fertilization, embryonic growth, and evolution and implantation (3). Obese women exhibit lower levels of sex hormone-binding globulin (SHBG) and higher levels of androgens, estrogens, and insulin than those with normal body weight (3, 23, 24). Owing to extensive adipose tissue in obese women and the high activity of aromatase enzyme, a larger amount of androgens is converted into estrogens (25), which in turn through negative feedback on the HPG axis impairs gonadotropin secretion resulting in ovulation dysfunction and irregular menstruation (3, 25).

Obesity also adversely affects the male reproductive system and decreases fertility rates (26, 27) owing to a decrease in sperm concentration, an increase in the number of abnormal sperm, and abnormal sperm motility (28, 29). The results of a meta-analysis showed that for every five units of BMI increase, all sperm parameters, including total sperm count, sperm concentration, and semen volume, declined by 2.4%, 1.3%, and 2.0%, respectively (30).

Maternal obesity and HFD also have adverse effects on the reproductive systems of offspring (18). Epidemiological studies in recent decades show that maternal nutrition during pregnancy has important effects on the offspring’s fertility (31). Maternal nutrition during pregnancy can lead to growth abnormalities and reproductive dysfunctions (32). Our previous study revealed that maternal HFD during pregnancy and lactation markedly decreased the number of primary, secondary, and graafian follicles, while increasing the number of atretic follicles in their offspring (18). HFD has also been shown to increase atretic follicles through up-regulation of ovarian cell cycle inhibitors, expansion of granulosa cells (Gc) apoptosis (33), or modifications of the expression of genes engaged in growth, development, and apoptosis of follicles in the ovary (34).

## 3 Diabetes and the reproductive system

The International Diabetes Federation estimates that 387 million people have diabetes and this is expected to rise to 592 million by 2035 (35, 36). The most common feature of diabetes is hyperglycemia, which occurs as a result of impaired insulin secretion or insulin action (37). Diabetes has become a major public health concern due to its complications, such as neuropathy, nephropathy, retinopathy, cardiovascular disease, and subfertility and infertility (38).

In recent decades, the link between obesity and T2DM has become well-known, and the main cause of this relationship is insulin resistance (39). Obesity is described by enlarged adipose tissue and dyslipidemia resulting in an excessive non-esterified fatty acids (NEFAs) release and the secretion of several biologically active elements, so-called adipokines, including tumor necrotic factor-α (TNF-α) and interleukin-6 (IL-6), leptin, and adiponectin, from the adipose tissue, ultimately promoting systemic low-grade inflammation or meta-inflammation. Obesity-associated inflammation can impair insulin signaling through serine phosphorylation of insulin receptor substrate-1 (IRS-1) and inhibiting phosphatidylinositol 3-kinase (PI3K)/protein kinase B (PKB) signaling, resulting in impaired glucose uptake. Besides, disproportionate FFA delivery to the liver increases the rate of gluconeogenesis, resulting in hyperglycemia. Furthermore, long-term exposure to elevated FFAs and increased hepatic triglyceride and glucose synthesis synergistically cause damage to the β-cells of the pancreas and impair insulin secretion, contributing to the development of insulin resistance and T2DM (40, 41).

Increasing the incidence of diabetes in both genders is a major concern for reproductive health (42). Clinical evidence and animal studies have proven that diabetes disrupts fertility, directly by impairing the function and structure of the gonads or indirectly by affecting the HPG axis (43–45). A low amount of gonadotropin-releasing hormone (GnRH) or a decrease in the sensitivity of the pituitary gland to GnRH is one of the factors related to the HPG axis dysfunction (46). The dopaminergic activity also increases in diabetic patients, which can subsequently inhibit GnRH secretion (46).

In the male reproductive system, diabetes induces structural changes like increased interstitial space, the destruction of germinal epithelium and abnormal pattern of seminiferous tubules (47), impairs glucose metabolism in Sertoli/blood testes barrier, reduces GnRH, gonadotropins, and testosterone levels, and the sensitivity of the pituitary gland to GnRH (46). Besides, diabetes decreases sperm quality and/or function, disrupts spermatogenesis, and results in ejaculatory dysfunction and a decreased libido (2, 47–50). Moreover, diabetes *via* decreasing serum testosterone and gonadotropins levels impairs the feedback mechanisms in Luteinizing hormone-releasing hormone (LHRH) producing cells (43, 44, 47, 50).

Women with diabetes may experience delayed menstruation and earlier menopause, which can affect fertility (51). Furthermore, the risk of polymenorrhea and amenorrhea significantly are increased in diabetic women compared to healthy women (52, 53). Type 1 diabetes can also reduce ovary size and oocyte quality (54), contribute to mitochondrial dysfunction during meiosis, and cause apoptosis of cumulus cells and DNA methylation (54). Besides, hyperinsulinemia observed in T2DM can change the levels of insulin-like growth factor binding protein (IGFBP), insulin-like growth factor 1 (IGF-1), and SHBG, which in turn cause an increase in androgen secretion from the ovaries and adrenals, and ultimately anovulation (46).

## 4 Gestational diabetes mellitus and the reproductive system

Obesity and overweight are also associated with a clustering of metabolic risk factors in early pregnancy and an increase in the risk of GDM (55). During the second and third trimester, 5-10% of pregnant women experience GDM, which involves hyperglycemia, glucose intolerance, and insulin resistance (56–58). Obesity, inactivity, advanced maternal age, family history of T2DM, GDM in the previous pregnancy and PCOS may cause GDM (59).

GDM has a series of negative effects on the health of the mother and offspring (58). Evidence shows that GDM increases fetal macrosomia and preeclampsia in the short term (58). It is estimated that 70% of women with GDM may develop T2DM later, up to 28 years after delivery (60). Infants born to mothers with GDM are more prone to neonatal hypoglycemia, hyperbilirubinemia, hypocalcemia, respiratory distress syndrome, and polycythemia (61). Moreover, GDM offspring will be at an increased risk of obesity and impaired glucose metabolism during childhood, adolescence, and youth (62).

Of note, diabetes adversely affects the reproductive function of offspring (63, 64). In this regard, offspring born to diabetic dams showed prostate tissue damage, decreased testosterone levels, and decreased sperm count and testicular tissue weight (65). Interestingly, GDM and intrauterine exposure to hyperglycemia predispose offspring to future reproductive dysfunction by altering the expression of genes involved in the differentiation and proliferation of Sertoli cells in the testes like *p27kip1, CX43* and aromatase (66).

## 5 Apelin: structure, receptor, and expression

Apelin is a small endogenous peptide, which interacts with class A G-protein-coupled receptor (GPCR), the APJ receptor. The *APLN* gene, located on chromosome Xq 25-26, translates to a 77 amino acids prepropeptide or apelin precursor, which is then cleaved into proapelin or apelin-55 through a proteolytic event. Proapelin is further cleaved into different apelin isoforms, based on their length, including apelin 13, 17, and 36. Apelin has a shot half-life (less than 5 min), and its plasma concentration in healthy human and mice is 0.26 ± 0.03 nmol/L and 100–1000 pg/mL, respectively (67). The APJ receptor has varying affinity for different apelin isoforms. Short isoforms like apelin-13 have low affinity and quickly dissociate, whereas longer isoforms (apelin-17 and -36) have high affinity and bind tightly. Since apelin-13 exhibits much stronger biological potency than apelin-36, it is widely used for exploring the physiological effects of apelin in *in vitro* and *in vivo* preparations (20). Post-translational modification of apelin-13 produces pyroglutamate [Pry (1)- apelin-13, containing a pyroglutamate group at its N-terminal, which is more resistant to degradation by peptidases and has a longer half-life and represents the major isoform of apelin in human tissues (68). The concentration of apelin isoforms is not the same in different organs. For example, apelin-36 is the most abundant form in the testis, uterus, and lung, and [Pry (1)]-apelin-13 is the dominant isoform in human blood (ranges from 7.7 to 23.3 pg/ml), brain, and heart (69). Moreover, pry^1^-apelin-13 and apelin-17 are the predominant forms in the plasma (70, 71).

The apelin receptor (APJ), an intron-less gene and a GPCR, was first identified in 1993. In humans, APJ is encoded by the *APLNR* gene, presented at chromosome 11q12, and in rats and mice, it is encoded by *Aplnr* gene, located on the chromosomal locations 3q24 and 2E1, respectively (13). Compared to the rat and mouse APJ, the human’s protein structure of apelin receptors contains 380-amino acid (versus 377-amino acid in rat and mouse APJ) and shares 90% sequence homology with rat and 92% homology with mouse APJ. The endogenous ligands for the APJ receptor exhibit nanomolar affinity, and 13-amino acid in their C-terminal region are essential for receptor binding (72).

Apelin and its receptor are expressed in many peripheral tissues in addition to the central nervous system, including the lungs, cardiovascular system, kidneys, mammary glands, white adipose tissue, gastric mucosa, testis, and uterus (73). In addition, studies in mice revealed that apelin is expressed in the endothelial part of the arterioles in the liver, pancreas, lung, adipose tissue, and spleen (74, 75). **Table 1** shows the expression pattern of Apelin/APJ receptor in various tissues and species.

**Table 1:** Apelin/APJ receptor expression pattern in various tissues and species.

	Tissues		Species	References
Nervous system	**Hypothalamus** (PVN, SON Magnocellular, Arcuate nucleus, Ventromedial nucleus), POMC **Pituitary lobes** (anterior, intermediate, posterior) Medulla oblongata Hippocampus Septum Amygdale Forebrain Brainstem Cerebellum Olfactory bulb Spinal cord Striatum Thalamus Cerebral cortex		Human, rat, mice, lizard	(1–8, 22)
Peripheral system	Non-Reproductive tissues	Plasma/serum Kidney Cardiovascular Skin Pancreas Spleen Lung and Bronchus Adipose tissue Liver Adrenal gland Skeletal muscle Thymus Stomach Thyroid glands Intestine Eye Bladder Mammary gland	Human, rat, mice, lizard Rat, mice, human, lizard Human, rat, mice, rabbit, lizard, zebrafish Human, lizard Rat; mice, human, lizard Mice, human, lizard Human, rat, mice, lizard, canine Human, rat, mice Rat, mice, human, lizard Human, rat, mice, lizard Human, rat, mice, lizard Human, mice Mice, rat, bovine, lizard Rat, lizard Rat, mice Monkey Mice, rat Sheep, rat	(9–14) (15–21)
	Reproductive tissues	**Placenta** **Uterus** **Ovary** (Gc, Cumulus, Tc, oocytes, zona pellucida, antral follicles) **Corpus luteum** **Testis** (Spermatids, Spermatozoa, Residual bodies, Leydig cells, Sertoli cells, Seminiferous tubules) **Embryo**	Human, mice, rat Mice, rat, human Human, mice, rat, sheep, monkey, porcine Human, rat, mice, bovine, buffalo, porcine Zebrafish, frog	(23) (24) (25) (22, 26–28) (29, 30) (31) (32) (33) (29, 34–36) (37) (38)

APJ couples to different G-protein subunits, including G_αi/o_, G_αs_, G_αq/11_, and G_α12/13_. The APJ receptor couples to G_αi/o_ G-protein and subsequently inhibits adenylated cyclase and c-AMP production thereby the protein kinase A (PKA) pathway, and activates extracellular-regulated kinases (ERKs) and phosphoinositide 3-kinase (PI3K)/Akt (PKB) signaling, which play a fundamental role in cell proliferation and survival. Conversely, G_αs_ mediates the activation of adenylyl cyclase, causing PKA production. APJ coupling to Gαq/11 activates the phospholipase C (PLC)/AMP-activated protein kinase (AMPK) pathway, leading to increased intracellular calcium. Furthermore, activation of G_α12/13_ results in rearrangement of the cytoskeleton (76). Apelin-13 is a potent Gα_i1_ activator and promotes cell proliferation, migration, and survival, and metabolic function *via* activation of the PI3K/Akt or mitogen-activated protein kinase (MAPK) pathways (77). All bioactive apelin isoforms bind to APJ to couple G_αi_ and inhibit cAMP production. Both apelin-13 and apelin-36 stimulate APJ coupling to G_αi_ and G_αq_, resulting in the activation of ERK1/2 and PLC signaling pathways (78).

Upon sustained activation of APJ, phosphorylation of APJ receptor by GPCR kinase-β recruits β-arrestin promoting receptor desensitization and internalization (79). The fate of the internalized APJ receptor is ligand-dependent, in which [Pry (1)]-apelin-13 or apelin-13 internalized receptor is rapidly detached from β-arrestin and recycled to the cell membrane, whereas apelin-36 forms a stable binding of β-arrestin to APJ and leads to its degradation by lysosome (80).

## 6 Central effects of apelin

Apelin and its receptor are widely distributed in neurons and oligodendrocytes of the different brain regions including the hypothalamus, medulla oblongata, hippocampus, septum, amygdale, forebrain, and brainstem (12), indicating that the apelinergic system plays a major role in neural signaling (73). The protective effect of apelin on cortical neurons can be exerted by inhibiting the production of reactive oxygen species (ROS), the release of cytochrome *c* from mitochondria into the cytosol, mitochondrial depolarization, and apoptosis *via* Erk/Akt signaling pathways (81). In a study that examined the effects of apelin-13 on inflammation caused by cerebral ischemia-reperfusion injury in rats, it was shown that treatment with apelin-13 at the beginning of reperfusion decreased the expression of inflammatory mediators such as IL-1β, TNF-α, and intercellular adhesion molecule-1 (ICAM-1) (82). The apelin receptor is also expressed in the corticotrophs in the pituitary gland, where it stimulates the release of ACTH in an autocrine/paracrine way (83).

The action of apelin on the body depends on the dose and route of administration (84). Administration of apelin intracerebroventricularly (i.c.v.) in a dose-dependent manner, increases the secretion of corticotropin-releasing hormone (CRH), adrenocorticotropic hormone (ACTH), corticosterone, vasopressin, while decreases the secretion of prolactin, thyroid-stimulating hormone (TSH), growth hormone, FSH, and LH (83, 85). It has been shown that blood pressure, nutritional behavior, and the secretion of pituitary hormones can be altered by intraventricular injection of apelin in mice (85).

Apelin is also involved in regulating food consumption and appetite. Apelin suppresses appetite by inducing α-melanocyte-stimulating hormone (α-MSH) release expression in pro-opiomelanocortin (POMC) neurons (86). Taheri et al. reported that intraventricular injection of apelin to fed animals had no significant effect on their food intake; however, apelin administration (at a dose of 30 nmol) in the fasted animals increased food intake at 2-4 h (85).

Previous studies showed that apelin-containing neurons in the hypothalamic nuclei, namely paraventricular (PVN) and supraoptic (SON) project toward the posterior pituitary and release apelin along with vasopressin (VP) and oxytocin into the blood, indicating that apelin affects fluid homeostasis and drinking behavior, but still many inconsistencies exist that remain to be elucidated.

Apelin and APJ receptors are co-localized with VP in magnocellular VP-ergic neurons of the hypothalamus, where apelin inhibits the activity of these neurons and thereby hypothalamic VP release (87, 88). Preclinical and clinical evidence also confirms the reciprocal interaction between apelin and VP. Apelin not only reduces central VP secretion but also opposes the actions of VP on the kidney and inhibits water reabsorption in the renal collecting duct by preventing aquaporin 2 (AQP-2) channels translocation to the apical membrane (89, 90). Roberts et al. also showed that APJ receptor knockout impaired drinking behavior and water hemostasis, exhibited by decreased water intake and failure to concentrate urine in response to exposure to water restriction (91). In contrast, Kubra et al. demonstrated that apelin gene knockout had no effect on water intake in water-deprived mice (92).

In physiological conditions, magnocellular neurons of the hypothalamus balance apelin and VP release to maintain normal plasma osmolality. In healthy individuals, hyperosmolality increases VP and decreases apelin, resulting in water retention, while water loading induces opposite effects (70). Systemic or central apelin injection had different effects on water intake in animals. Some studies found that injecting apelin systemically or centrally in normovolemic animals increased water intake (85, 93), while others found that injecting apelin centrally in animals with HFD-induced obesity or water deprivation decreased water intake and VP plasma levels (94). Mitra et al. discovered that administering apelin centrally or peripherally to animals with free water access did not affect water intake and did not reduce water intake in water-deprived rats (95).

Central injection of apelin-17 to lactating mice attenuated plasma VP levels and increased diuresis (71). One study showed that subcutaneous administration of an apelin-17 stable analog (LIT01-196) in normal water-loading and normonatremic condition promoted aquaretic effects and increased urine output and water intake independent of affecting central VP secretion. Moreover, apelin-17 analog administration in the excessive VP secretion model (syndrome of inappropriate antidiuresis) corrected fluid homeostasis and plasma sodium levels by inhibiting the effect of VP on AQP-2 channels in the collecting duct and increasing water diuresis (96).

Nevertheless, intravenous injection of [Pry (1)]-apelin 13 to hydrated sheep has been shown to increase plasma VP concentration (97). An *in vitro* study also demonstrated that apelin-13 (100 nmol) stimulated the release of VP from hypothalamic explants. It seems that depending on the dose and isoform of apelin and the conditions of the experiment (physiological or special conditions such as water deprivation, lactation, and hyponatremia, which can change the basal secretion of VP) the effects of apelin on magnocellular VP-ergic neurons can be different (71, 85).

## 7 Peripheral effects of apelin

Previous reports support the effects of apelin on the cardiovascular system (98). Administration of apelin has been shown to increase the production of nitric oxide (NO) in the endothelium of blood vessels primarily through the activation of endothelial nitric oxide synthase (eNOS) and thus decreases blood pressure (74). Furthermore, apelin exerts an antioxidant effect in the cardiomyocytes, vessels, and adipocytes by suppressing the release of reactive oxygen species (ROS) and enhancing the expression of antioxidant enzymes (99, 100). The apelinergic system also stimulates gastric and endothelial cell proliferation, regulates catecholamine-mediated lipolysis, increases glucose uptake in insulin-sensitive tissues, promotes retinal angiogenesis, acts as a positive inotrope, and maintains fluid homeostasis (16, 101). Apelin activates Na+/H+ and Na+/Ca2+ exchangers and inositol triphosphate receptors, causing calcium release from the sarcoplasmic reticulum and increasing myocardial contraction (68, 102, 103).

## 8 Metabolic effects of apelin

### 8.1 Apelin and glucose metabolism

Apelin and its receptor, APJ, are distributed in the pancreatic tissue and contribute to the control of glucose metabolism (104, 105). The apelinergic system aids glucose homeostasis by enhancing glucose absorption in the gastrointestinal tract, boosting glucose transport in skeletal muscles and adipose tissues, and modulating insulin secretion (106). Previous studies proved that apelin dose-dependently modulates pancreatic insulin secretion, where a low dose of apelin-36 (2 nmol/kg) declines whilst a high dose (1 µM) of apelin-36 increases insulin release following intravenous glucose injection (107, 108). Apelin-13 also inhibits insulin secretion stimulated by high glucose concentrations (10 mM) as well as glucagon-like peptide-1 (GLP-1)-enhanced insulin secretion in insulinoma cells (109). In normal and insulin-resistant mice, injecting a low apelin-13 concentration (200 pmol/kg) enhanced insulin sensitivity and glucose uptake in skeletal muscle and adipose tissue (104).

The AMP-activated protein kinase (AMPK), an energy sensor, stimulates the absorption of glucose in skeletal muscle, oxidizes fatty acids in adipose tissue, and reduces the production of liver glucose (110). Yue et al. (2010) indicated that apelin boosts glucose transport in C2C12 muscle cells by activating the AMPK pathway, and apelin gene knockout in high fat- and- carbohydrate-fed mice decreases insulin sensitivity (105). Apelin also stimulates glucose transport in human fat tissue through the activation of the AMPK pathway (111). Additionally, apelin increases insulin-related glucose uptake in insulin-resistant 3T3-L1 fat cells *via* activation of the PI3K/Akt pathway (112). Administration of [Pry (1)]-apelin 13 improves myocardial glucose uptake by enhancing the translocation of GLUT4 in an AMPK-reliant way (69, 113).

Moreover, apelin levels in insulin-resistant disorders, namely obesity, and T2DM, are increased, and apelin treatment increases insulin sensitivity, glucose tolerance, and fatty acid oxidation (114, 115), representing the effectiveness of exogenous apelin on diabetes-related complications.

Previous studies reported that oral glucose administration quickly increased the secretion of apelin by intestinal epithelial cells, consequently, apelin increases glucose absorption from the intestine by activation of the AMPK pathway and increasing glucose transporters in the brush border membrane, causing an increase in glucose levels in the portal vein and rapid secretion of insulin from the pancreas (116, 117). On the other hand, an oral APJ antagonist administration before glucose gavage reduces glucose absorption by enterocytes and reduces hyperglycemia (116). An *in vitro* study also revealed that human endothelial cultured cells secrete apelin in response to glucose (118).

Apelin is also expressed in the hypothalamus and regulates glucose homeostasis in the central nervous system (CNS) in an eNOS-dependent manner. Injecting apelin-13 i.c.v. at low doses reduced blood glucose levels, while high doses increased blood glucose and insulin levels, and decreased insulin sensitivity in normal diet-fed animals, possibly by over-stimulating the sympathetic nervous system and enhancing liver glycogen breakdown and gluconeogenesis (119, 120). Central injection of apelin in obese diabetic mice induced hyperglycemia, while there was a slight change in insulin level (119).

### 8.2 Apelin and lipid metabolism

Apelin also influences lipid metabolism in both isolated adipocytes and differentiated 3T3-L1 adipocytes. Apelin inhibits isoproterenol (β-adrenergic agonist)-induced lipolysis *via* a pathway, including Gq, Gi, and AMPK (121). In insulin-resistant adipocytes, apelin increases the amount of perilipin around lipid vacuoles and enhances the activity of the AMPK pathway, hence increasing lipid solidity and resistance to lipase function (122). In obese and insulin-resistant mice, chronic apelin treatment increases fatty acid oxidation in muscles by activating the AMPK pathway (114).

*In vivo* studies have indicated that the expression of apelin is strongly regulated by nutritional status (123). Fasting has been shown to suppress apelin expression while re-feeding returns apelin level to normal (111). Yang et al. found that 20 weeks of HFD resulted in increased apelin levels and gene expression of its receptor in adipose tissue and gastrocnemius muscle

(124). Garcia-Diaz et al. also reported that 50-day HFD increases apelin mRNA in the subcutaneous adipose tissue (125). HFD feeding during pregnancy and lactation can also increase serum levels of apelin in dams while decreasing serum apelin in adult male offspring (19, 123, 126). Paternal HFD exposure affects offspring’s metabolic traits *via* epigenetic changes in *leptin* and *adiponectin* gene promoters, though to a lesser extent than *in utero* HFD exposure (127). Our previous study also showed that maternal HFD increased serum levels of apelin-13 and its receptor gene expression in the ovarian tissue of offspring (18). Moreover, maternal nutritional status can affect breast milk apelin levels. In this regard, a study showed that HFD feeding during lactation increases the concentration of apelin in breast milk, possibly by up-regulation of apelin expression in myoepithelial cells in the mammary gland (126). Long-term HFD consumption by inducing insulin resistance and hyperinsulinemia increase apelin secretion from adipose tissue (123, 128, 129).

## 9 Apelin in metabolic disorders

### 9.1 Obesity

Obese patients have increased apelin levels and APJ receptor expression in adipose tissue (130, 131), while weight loss reduces plasma apelin levels (132). Moreover, in experimental obesity models that are associated with hyperinsulinemia, apelin levels significantly increased (16, 133). It seems that high levels of fatty acids reduce cell sensitivity to insulin and eventually cause insulin resistance and hyperinsulinemia, which increases apelin secretion from adipose tissue (134). On the other hand, apelin-transgenic mice failed to gain body weight under a HFD feeding condition for 20 weeks and showed decreased body adiposity and resistance to diet-induced obesity (135). Moreover, apelin-deficient mice showed decreased plasma adiponectin, increased insulin, impaired glucose and insulin tolerance, and insulin resistance (105). [Pry (1)]-apelin-13 infusion over 4 weeks improved insulin sensitivity and decreased insulin levels in apelin-null and *db/db* mice (105).

Adiponectin is an adipokine secreted by adipose tissue, which increases glucose uptake and insulin sensitivity and lessens hepatic gluconeogenesis. Adiponectin levels and insulin sensitivity are positively correlated, but inversely proportional to body fat and leptin levels. Obesity often leads to reduced adiponectin levels and insulin resistance (136, 137). Besides, the adiponectin/leptin ratio is a suggestive indicator of metabolic abnormalities and insulin resistance (138). Another study showed that chronic apelin-13 administration decreased white adipose tissue weight and body weight gain, increased energy expenditure, decreased leptin, and increased adiponectin levels in serum, but there was no change in food intake (139).

Apelin treatment for 28 days improved insulin sensitivity and skeletal muscle lipid oxidation and utilization, without significantly affecting body weight in obese and insulin-resistant rats (114). Another study showed that chronic apelin treatment (0.1 μmol/kg/day for 28 days) in HFD-induced obese mice decreased glucose and insulin levels, and increased fatty acid oxidation and mitochondrial biogenesis in soleus muscle (140). Therefore, optimal levels of apelin in blood circulation can probably be effective in delaying or reducing insulin resistance.

### 9.2 Diabetes

Emerging evidence supports the role of apelin in the pathogenesis of diabetes (141). Several studies have demonstrated an increase in plasma concentrations of apelin in patients with type 1 or type 2 diabetes (131). Apelin regulates insulin secretion, glucose uptake, lipid oxidation, apoptosis, oxidative stress, and angiogenesis, playing a critical role in diabetes-related complications like cardiovascular diseases, diabetic nephropathy, and endothelial dysfunction (142). Feng et al. found that chronic apelin-13 treatment (0.1 μmol/kg for 10 weeks) in T2DM improved insulin sensitivity and protect pancreatic beta cells (143). In the streptozotocin-induced type 1 diabetes model, decreased insulin levels is accompanied by a decrease in apelin levels (16, 133). Insulin regulates apelin expression and secretion *via* the PI3K/protein kinase C/MAPK pathways in mice and humans (16). In fact, hyperapelinemia is a compensatory mechanism that inhibits pancreatic secretion and increases insulin sensitivity and glucose absorption in mouse muscle tissues in a non-insulin-dependent manner (144).

In addition, acute administration of apelin-13 (100 μl/2 min) can improve glucose tolerance and increase glucose utilization in healthy and insulin-resistant mice by activating the AMPK and Akt signaling pathways (145). In type 1 diabetes, apelin therapy (400 pmol/kg) for 10 weeks enhanced pancreatic islet mass, insulin content, and reduced endoplasmic reticulum stress in the pancreatic islets (146). Apelin-13 was reported to improve glucose metabolism, dyslipidemia, insulin sensitivity, and decrease leptin levels in an HFD-induced T2DM model (147).

### 9.3 GDM

GDM is a complication related to glucose intolerance (148). In a healthy pregnancy, insulin sensitivity decreases during pregnancy to maintain glucose for fetal consumption, but in most women, due to the production of sufficient amounts of insulin by the pancreas, blood glucose levels remain normal during pregnancy (58). In GDM, the pancreas is unable to produce enough insulin, and blood glucose levels remain at a high level (58). Although hyperglycemic conditions improve after delivery, GDM can increase the risk of postpartum T2DM (58). Mayeur et al. demonstrated that the apelin/APJ system is involved in the control of glucose homeostasis in the fetus and infant. They showed that administration of apelin in embryonic day 17 increased the transplacental transfer of glucose by fetal tissues. Moreover, injection of apelin at doses of 10 and 15 nmol/kg in neonates reduced blood glucose, while higher doses of apelin (20 and 40 nmol/kg) increased blood glucose and decreased insulin levels in neonates (149).

Several studies have also investigated apelin concentration in pregnant women with GDM; however, there is heterogeneity in the reported results. Although some studies reported decreased levels of apelin in GDM women compared to the control group (150–153), Sun et al. in a systematic review and meta-analysis demonstrated no significant change in serum apelin in pregnant women with GDM (154). In contrast, Aslan et al. reported higher serum apelin levels in pregnant women with GDM than in healthy pregnant women (155). Also, a recent meta-analysis showed that GDM is associated with increased apelin levels (156). Moreover, Boucher et al. found increased apelin levels at the beginning of pregnancy in obese women and mice (157).

## 10 Apelin and the HPG axis and reproductive system

### 10.1 HPG axis

The function of the gonads, including the ovaries and testes, is regulated by the HPG axis (158). This axis comprises 3 parts, including the hypothalamus, which is responsible for synthesizing GnRH, the pituitary gland, where LH and FSH are synthesized, and gonads, involved in the production of sex steroids and other hormones (158). Evidence shows that apelin and its receptors are widely expressed in the hypothalamus nuclei, namely PVN and SON, where GnRH is released (13, 159–161), and in all three pituitary lobes (anterior, intermediate, posterior) of rats (73), representing that apelinergic system is involved in the control of behavioral processes, energy homeostasis, and endocrine function namely reproductive function (162, 163). APJs are also expressed in the ovaries and testes and may play a role in regulating reproduction through the HPG axis (21). An earlier study reported that a single central injection of apelin-13 in rats activated the hypothalamic-pituitary-adrenal (HPA) axis and increased ACTH and corticosterone levels, whereas suppressed the pituitary hormones, indicated by diminished circulatory prolactin, LH, and FSH levels (85). Chronic i.c.v injections of apelin-13 for seven days in male rats significantly reduced LH release, testosterone levels, and the number of Leydig cells, while had no significant effects on FSH levels (21).

An *in vitro* study showed that the administration of progesterone and LH increased the levels of apelin and APJ mRNA in the Gc and theca cells (Tc), respectively (164). Tekin et al. showed that intraperitoneal chronic injections of apelin-13 (1, 5, and 50 µg/kg for 14 days) decreased LH, FSH, and testosterone levels, but had no significant effects on GnRH levels, indicating that its inhibitory effect on reproductive function is mediated mainly through suppression of pituitary hormones (165). Recently, it has been reported that activation of *APLNR* (apelin receptor gene) in GnRH-releasing embryonic stem cells protects the neurons against oxidative stress and apoptosis and increased cell proliferation in an Akt signaling-dependent manner. However, prolonged overexpression of complete blockage of the APJ receptor reduced GnRH release (166).

A study in pregnant rats showed increased apelin levels at the end of pregnancy (gestational day 21), and apelin administration dose-dependently strengthens the myometrial contractility of the uterine (167). Conversely, an *in vitro* study on human myometrial fibers demonstrated the inhibitory effect of apelin treatment (1 nmol/L to 1 μmol/L) on spontaneous and oxytocin-triggered isometric myometrial contractions (168). Likewise, Asalah et al. found that apelin (100 nmol/L) administration to isolated uterine strips of pregnant rats inhibited spontaneous uterine reactivity (169). Pregnant women with obesity exhibited increased apelin levels which causes a decrease in frequency and strength of myometrial contractions (170).

### 10.2 Functions of apelin in the ovary

Apelin and its receptor APJ are expressed in the ovaries of many species, such as bovine, buffalo, primates, porcine, rodents, and humans (18, 20, 171, 172). Apelin and APJ genes are expressed in different cells of the ovary. Shimizu et al. showed that the apelin gene is expressed only in the Tc of bovine follicles, while APJ gene is expressed both in the Tc and Gc (164). In human ovarian cells, apelin and its receptor are expressed in the Gc, cumulus, Tc, and oocytes at different phases of follicular development (173).

Moreover, different reproductive hormones can affect the expression of apelin or its receptor in the ovary. An *in vitro* study revealed that FSH induced apelin/APJ expression in the Gc, whilst in the Tc, LH stimulated the expression of both apelin and APJ receptors, and progesterone prompted the expression of APJ mRNA in bovine follicles (164). Also, the level of apelin is different at the physiological stages of the ovulation cycle and pregnancy (171). In the bovine ovary, apelin mRNA and APJ receptors are increased during the early and mid-luteal stages of the estrus cycle, while following corpus luteum (CL) regression, there is a decrease in their expression levels (171).

Apelin and its receptor are involved in the physiological functions of ovarian cells, including steroidogenesis, folliculogenesis, proliferation, and apoptosis (164, 172–174). Previous *in vitro* studies on human, bovine, and porcine ovarian cells showed that apelin improved follicles proliferation and development and survival of Gc *via* activation of the ERK1/2 and Akt signaling pathways. Additionally, apelin increased the secretion of progesterone and estradiol (E2) and increased the activity of enzymes catalyzing the synthesis of steroids, including 3β-hydroxysteroid dehydrogenase/Δ^5–4^ isomerase (3β-HSD) and CYP19A1, by activating of the MAPK/AMPK pathway (173–176). Apelin also regulates the proliferation and apoptosis of ovarian cells (177). Apelin stimulated the PI3K/Akt signaling pathway to promote proliferation and inhibit apoptosis in the Gc (177). A recent *in vitro* study revealed that administration of apelin-13 to Gc of buffalo ovaries promoted IGF-1-induced progesterone synthesis but did not affect FSH-stimulated progesterone secretion, and boosted antioxidant capacity and Gc proliferation (178). Besides, the angiogenic effect of apelin is mediated through the stimulation of the proliferation and migration of endothelial cells. Apelin/APJ is expressed in the smooth muscle cells of bovine arterioles of the CL and controls the luteolysis process in the CL by inducing blood vessels expansion possibly due to the activation of the eNOS pathway and nitric oxide production (179, 180).

### 10.3 Apelin in ovarian pathologies

Apelin is linked to frequent female reproductive pathological conditions such as PCOS, ovarian cancer, and endometriosis (14, 181, 182). In PCOS patients, high levels of apelin in the blood and follicular fluid, and Gc of ovaries were reported (22, 183). Roche et al. reported that the expression levels of apelin and APJ in human Gc from obese PCOS are higher than in healthy women and non-obese patients (173). Moreover, administration of apelin-13 and 17 to primary human Gc increased IGF-1, estradiol, and progesterone secretion, as well as 3β-HSD protein expression. Sun et al. also showed higher apelin levels in PCOS patients than in control, which was positively correlated with BMI, insulin levels, and insulin resistance index (184). Nevertheless, Chang et al. reported lower serum apelin in non-obese PCOS subjects than in the control group (185). A study reported lower levels of apelin-36, apelin-12, LH, SHBG, and adiponectin and higher levels of leptin in obese PCOS patients. Besides, plasma levels of apelin isoforms were inversely correlated with leptin and LH, whereas apelin levels were positively associated with serum adiponectin levels (186). Mishra et al. demonstrated a decrease in adiponectin and an increase in leptin levels in the plasma of PCOS patients (187). Since leptin and adiponectin modulate steroidogenesis, gonadotrophins release, and fertility (8, 9), apelin replacement may improve reproductive dysfunction associated with obesity or T2DM through increasing adiponectin and the adiponectin/leptin ratio.

**Figure 1:**
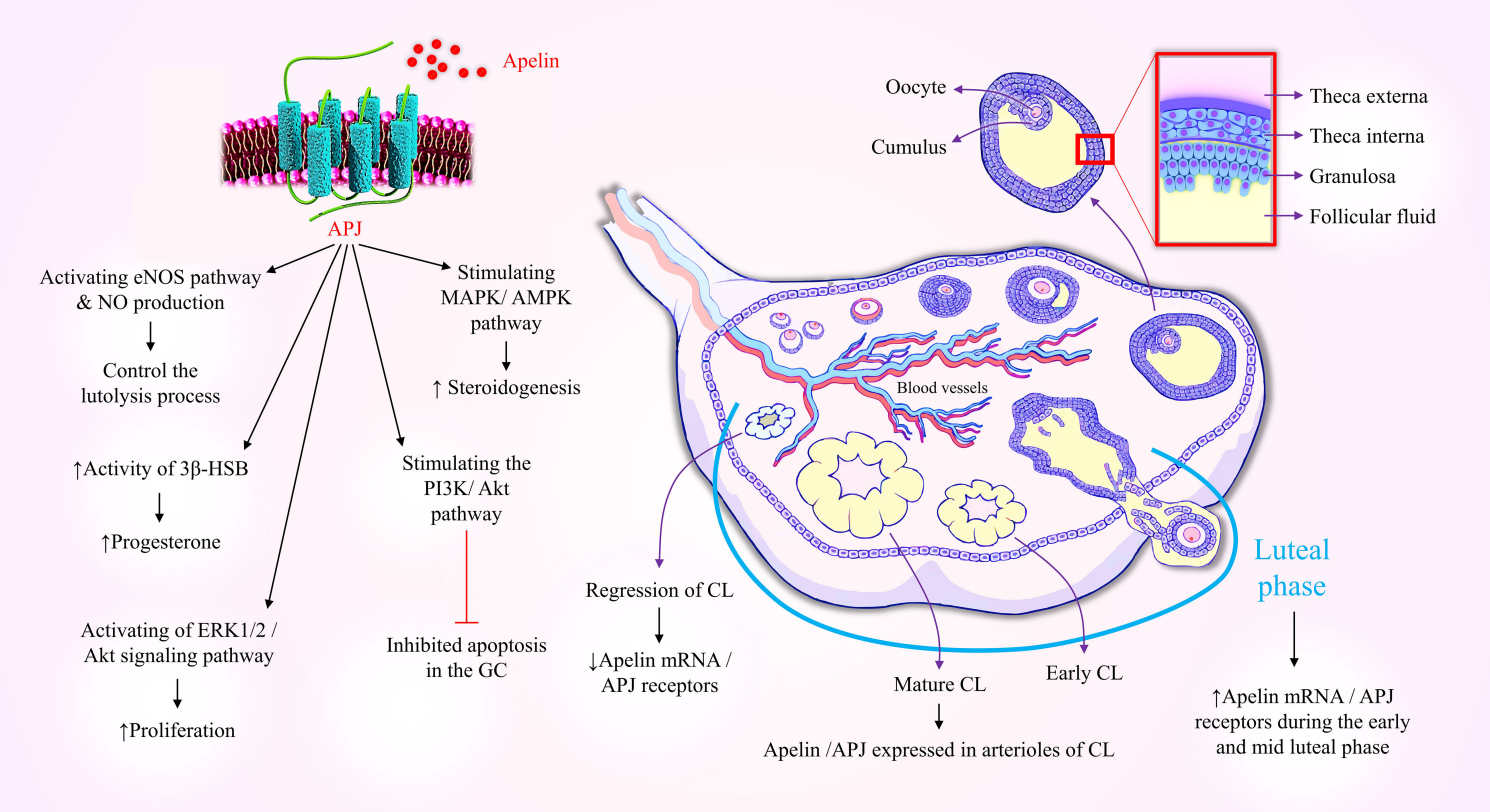
Schematic diagram indicating the involvement of apelin in ovarian physiology. Gc, Granulosa Cells; Tc, Theca Cells; APJ, Apelin receptor; CL, Corpus Luteum; eNOS, Endothelial nitric oxide synthase; NO, Nitric Oxide; PI3K, phosphatidylinositol 3-kinases; AKT, Protein kinase B; MAPK, Mitogen-Activated Protein Kinase; AMPK, AMP-activated protein kinase; 3β-HSD, 3β-Hydroxy Steroid Dehydrogenase.

Apelin is also highly expressed in glandular cells of the ectopic and eutopic endothelium of women with endometriosis during the secretory phase (14). Recent studies also demonstrated a relationship between apelin and ovarian cancer (181, 188). APJ was found to be highly expressed in human ovarian tumor cells, and its activation increased cancer cell growth and proliferation by triggering the STAT3 pathway, which is linked to a worse prognosis. However, inhibition of APJ receptor by ML221 suppressed the pro-metastatic phenotype of the cancer cells (188). Despite the fact that age advances down-regulated endogenous apelinergic system, which could speed up age-related physiologic declines (189–191), there is limited evidence concerning the changes in apelin or its receptor levels in reproductive tissue with aging. The deficiency of apelin and its receptor genes in female mice aged eight to nine months resulted in reduced expression of the LH receptor and inhibin-α in the ovaries, indicating an early onset of infertility and the aging of the reproductive system (192). **Figure 1** shows the function of apelin/APJ in the ovaries.

### 10.4 Functions of apelin in the testis

Apelin and APJ receptors are also expressed in the testicular tissue of several species, like humans, rodents, and canines (193–195). A recent study found that apelin and APJ are expressed in Leydig cells of rats (194). Troisi et al. reported that apelin is present in canine spermatids, spermatozoa, and residual bodies, as well as in the Leydig cells and seminiferous tubules (193). Estienne et al. demonstrated that intraventricular infusion of a high dose of apelin-13 (10 nmol) reduced the number of Leydig cells (7). This evidence highlights the critical role of apelin and APJ receptors in the male reproductive system (7, 196).

**Figure 2:**
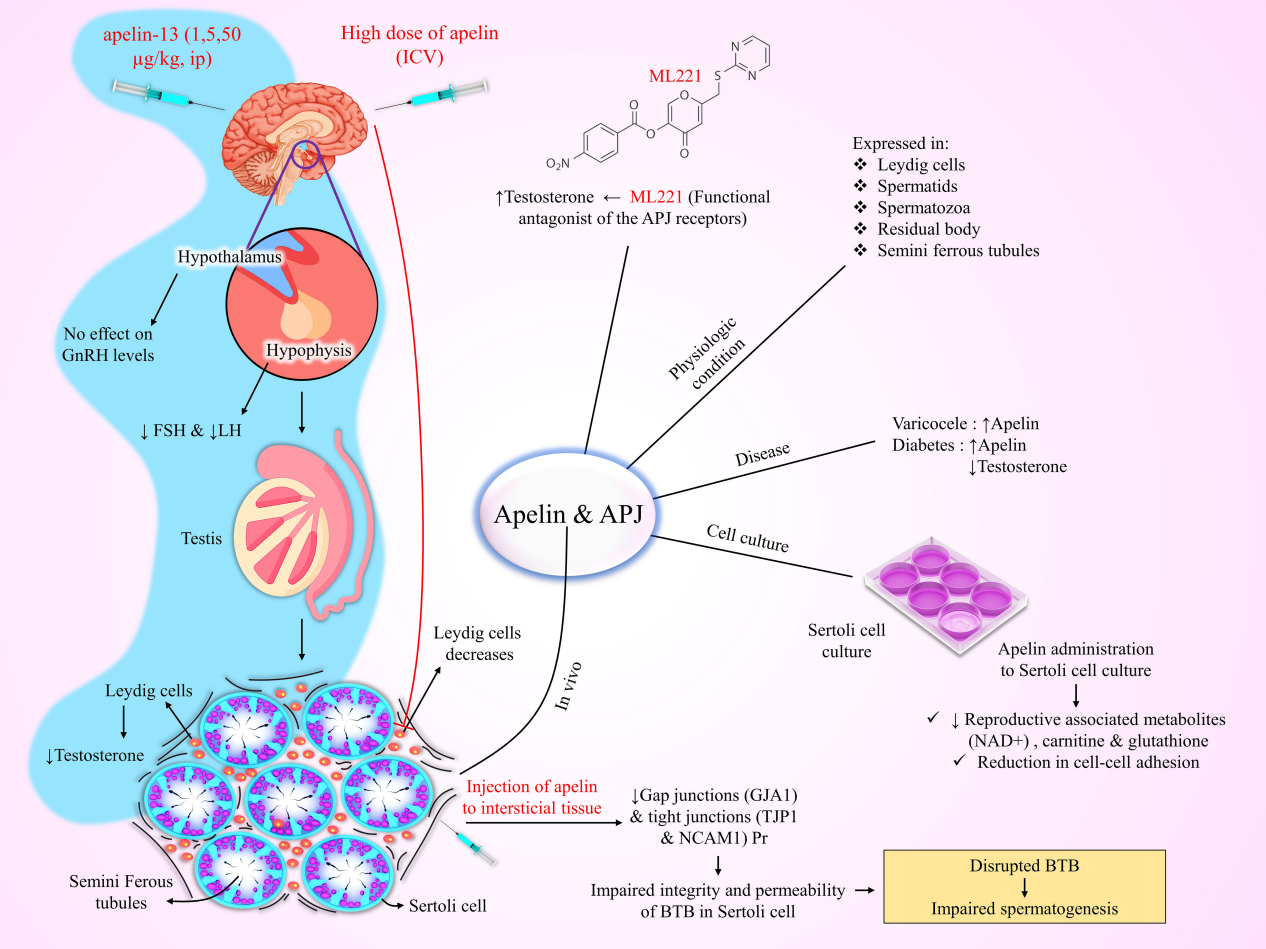
Schematic illustration of the expression of apelin in the testis and induction of infertility as a result of increased apelin in the male reproductive system. ICV, Intracerebroventricular; BTB, Blood-Testis Barrier.

Varicocele is an abnormal enlargement of scrotal veins, associated with inflammatory alterations in testicular tissue, and impairs spermatogenesis. Akkan et al. reported high levels of apelin in the testicular tissue of rats with varicocele, whereas APJ expression was decreased, possibly because of receptor internalization (197). Das et al. reported a direct relationship between increased apelin and APJ levels in the testicular Leydig and germ cells and decreased testosterone release in type 1 diabetic mice (198). Moreover, in an *in vitro* examination, they showed that the apelin receptor antagonist, ML221, increased testosterone synthesis, while apelin-13 had no effect on testosterone secretion in diabetic testis (198). Interestingly, Song et al. found that protein expression of apelin is increased in the testis of diabetic mice and human cell culture, while there was no significant change in APJ protein levels (199). Moreover, apelin injection into the testicular interstitium of diabetic mice impaired the integrity and permeability of the blood-testis barrier (BTB) in Sertoli cells by decreasing gap junction and tight junction protein levels, suggesting that abnormally elevated apelin levels impair spermatogenesis by disrupting the BTB (199). Conversely, the APJ antagonist, ML221, restored BTB integrity, and improved blood testosterone levels, sperm concentration, and motility, but did not restore natural fertility in diabetic mice (199). Additionally, apelin administration to the Sertoli cell culture decreased reproductive-associated metabolites, including β-nicotinamide adenine dinucleotide (NAD^+^), carnitine, and glutathione, while increased in the amount of palmitelaidic acid (198, 199). Based on the above, targeting the apelinergic system holds a promising approach to improve male reproductive function and fertility in diabetic conditions. **Figure 2** summarized the functions of the apelinergic system in the male reproductive system.

## 11 Conclusion

The apelinergic system is an essential regulator of energy metabolism and exerts diverse beneficial effects against the development of the metabolic disorders, particularly diabetes and obesity. Apelin also regulates fertility and reproductive functions in physiological and pathological conditions through its autocrine and/or paracrine effects. Therefore, targeting this pathway is a current demand and a novel outlook that could provide more treatment options for improving reproductive capacity in metabolic disorders. More detailed studies are required to address all roles of the apelinergic system in this context.

## Author contributions

KM and ZZ wrote the first draft of the manuscript. FF conceived and designed the study. FF, GH, and SN reviewed and edited the manuscript. All authors read and approved the final version of the manuscript.

## References

[1] Godfrey KM Gluckman PD Hanson MA. Developmental origins of metabolic disease: life course and intergenerational perspectives. Trends Endocrinol Metab (2010) 21(4):199–205. doi: 10.1016/j.tem.2009.12.008 20080045

[2] Niwas Jangir R Chand Jain G. Diabetes mellitus induced impairment of male reproductive functions: a review. Curr Diabetes Rev (2014) 10(3):147–57. doi: 10.2174/1573399810666140606111745 24919656

[3] Amiri M Tehrani FR. Potential adverse effects of female and male obesity on fertility: a narrative review. Int J Endocrinol Metab (2020) 18(3):e101776. doi: 10.5812/ijem.101776 33257906 PMC7695350

[4] Macedo ICD Medeiros LF Oliveira CD Oliveira CMD Rozisky JR Scarabelot VL. Cafeteria diet plus chronic stress alter leptin serum level and specific adipose tissue weights in six weeks of treatment. Rev HCPA Porto Alegre (2012) 123(1-2):90–7. doi: 10.1016/j.peptides.2012.08.007

[5] Kanoski SE Davidson TL. Western Diet consumption and cognitive impairment: links to hippocampal dysfunction and obesity. Physiol behavior (2011) 103(1):59–68. doi: 10.1016/j.physbeh.2010.12.003 PMC3056912 21167850

[6] Wójcik M Chmielewska-Kassassir M Grzywnowicz K Woźniak L Cypryk K. The relationship between adipose tissue-derived hormones and gestational diabetes mellitus (GDM). Endokrynologia Polska (2014) 65(2):134–42. doi: 10.5603/EP.2014.0019 24802737

[7] Estienne A Bongrani A Reverchon M Ramé C Ducluzeau P-H Froment P. Involvement of novel adipokines, chemerin, visfatin, resistin and apelin in reproductive functions in normal and pathological conditions in humans and animal models. Int J Mol Sci (2019) 20(18):4431. doi: 10.3390/ijms20184431 31505789 PMC6769682

[8] Singh A Choubey M Bora P Krishna A. Adiponectin and chemerin: contrary adipokines in regulating reproduction and metabolic disorders. Reprod Sci (2018) 25(10):1462–73. doi: 10.1177/1933719118770547 29669464

[9] Choubey M Ranjan A Bora PS Baltazar F Martin LJ Krishna A. Role of adiponectin as a modulator of testicular function during aging in mice. Biochim Biophys Acta (BBA)-Molecular Basis Disease (2019) 1865(2):413–27. doi: 10.1016/j.bbadis.2018.11.019 30471430

[10] Newson MJ Roberts EM Pope GR Lolait SJ O’Carroll A-M. The effects of apelin on hypothalamic–pituitary–adrenal axis neuroendocrine function are mediated through corticotrophin-releasing factor-and vasopressin-dependent mechanisms. J endocrinology (2009) 202(1):123. doi: 10.1677/JOE-09-0093 19395447 PMC2695660

[11] Roberts E Pope G Newson M Landgraf R Lolait S O’Carroll AM. Stimulus-specific neuroendocrine responses to osmotic challenges in apelin receptor knockout mice. J neuroendocrinology (2010) 22(4):301–8. doi: 10.1111/j.1365-2826.2010.01968.x 20136689

[12] Reaux A Gallatz K Palkovits M Llorens-Cortes C. Distribution of apelin-synthesizing neurons in the adult rat brain. Neuroscience (2002) 113(3):653–62. doi: 10.1016/S0306-4522(02)00192-6 12150785

[13] O’Carroll A-M Lolait SJ Harris LE Pope GR. The apelin receptor APJ: journey from an orphan to a multifaceted regulator of homeostasis. J Endocrinol (2013) 219(1):R13–35. doi: 10.1530/JOE-13-0227 23943882

[14] Ozkan ZS Cilgin H Simsek M Cobanoglu B Ilhan N. Investigation of apelin expression in endometriosis. J Reprod Infertility (2013) 14(2):50. PMC3719321 23926564

[15] Gören K Sağsöz N Noyan V Yücel A Çağlayan O Bostancı MS. Plasma apelin levels in patients with polycystic ovary syndrome. J Turkish German Gynecological Assoc (2012) 13(1):27. doi: 10.5152/jtgga.2011.74 PMC3940220 24627671

[16] Boucher JRM Masri B Daviaud D Sp G Guigné C Mazzucotelli A. Apelin, a newly identified adipokine up-regulated by insulin and obesity. Endocrinology (2005) 146(4):1764–71. doi: 10.1210/en.2004-1427 15677759

[17] Bełtowski J. Apelin and visfatin: unique” beneficial” adipokines upregulated in obesity? Med Sci monitor: Int Med J Exp Clin Res (2006) 12(6):RA112–9. 16733497

[18] Mehri K Khojasteh SMB Mahdi BKS Fereshteh F Oskuye ZZ Ebrahimi H. Effect of troxerutin on apelin-13, apelin receptors (APJ), and ovarian histological changes in the offspring of high-fat diet fed rats. Iranian J basic Med Sci (2019) 22(6):637. doi: 10.22038/ijbms.2019.34158.8123 PMC6570758 31231491

[19] Diba R Mohaddes G Bavil FM Farajdokht F Bayandor P Hosseindoost M. Protective effects of troxerutin on maternal high-fat diet-induced impairments of spatial memory and apelin in the male offspring. Iranian J Basic Med Sci (2018) 21(7):682. doi: 10.22038/IJBMS.2018.28170.6901 PMC6098955 30140406

[20] Kurowska P Barbe A Chmielińska J Dupont J Rak A. Apelin in reproductive physiology and pathology of different species: a critical review. Int J Endocrinol (2018) 2018:1–12. doi: 10.1155/2018/9170480 PMC6011052 29977292

[21] Sandal S Tekin S Seker FB Beytur A Vardi N Colak C. The effects of intracerebroventricular infusion of apelin-13 on reproductive function in male rats. Neurosci Letters (2015) 602:133–8. doi: 10.1016/j.neulet.2015.06.059 26149233

[22] Dravecká I Figurová J Lazúrová I. Is apelin a new biomarker in patients with polycystic ovary syndrome? Physiol Res (2021) 70(Suppl 4):S635. doi: 10.33549//physiolres.934708 35199548 PMC9054183

[23] Slopien R Horst N Jaremek JD Chinniah D Spaczynski R. The impact of surgical treatment of obesity on the female fertility. Gynecol Endocrinol (2019) 35(2):100–2. doi: 10.1080/09513590.2018.1500536 30599791

[24] Gaskins AJ. Recent advances in understanding the relationship between long-and short-term weight change and fertility. F1000Research (2018) 7:1–7. doi: 10.12688/f1000research.15278.1 PMC6206616 30416711

[25] Kasum M Orešković S Čehić E Lila A Ejubović E Soldo D. The role of female obesity on *in vitro* fertilization outcomes. Gynecol Endocrinol (2018) 34(3):184–8. doi: 10.1080/09513590.2017.1391209 29037105

[26] Sundaram R Mumford SL Buck Louis GM. Couples’ body composition and time-to-pregnancy. Hum reproduction (2017) 32(3):662–8. doi: 10.1093/humrep/dex001 PMC5400044 28158570

[27] Chambers TJ Anderson RA. The impact of obesity on male fertility. Hormones (2015) 14(4):563–8. doi: 10.14310/horm.2002.1621 26732149

[28] Tsao C-W Liu C-Y Chou Y-C Cha T-L Chen S-C Hsu C-Y. Exploration of the association between obesity and semen quality in a 7630 male population. PloS One (2015) 10(3):e0119458. doi: 10.1371/journal.pone.0119458 25822490 PMC4379020

[29] McPherson NO Lane M. Male Obesity and subfertility, is it really about increased adiposity? Asian J andrology (2015) 17(3):450. doi: 10.4103/1008-682X.148076 PMC4430951 25652636

[30] Guo D Wu W Tang Q Qiao S Chen Y Chen M. The impact of BMI on sperm parameters and the metabolite changes of seminal plasma concomitantly. Oncotarget (2017) 8(30):48619. doi: 10.18632/oncotarget.14950 28159940 PMC5564712

[31] Chavatte-Palmer P Al Gubory K Picone O Heyman Y. Maternal nutrition: effects on offspring fertility and importance of the periconceptional period on long-term development. Gynecol Obstet Fertil (2008) 36(9):920–9. doi: 10.1016/j.gyobfe.2008.06.020 18693060

[32] Jacobs S Teixeira DS Guilherme C da Rocha CF Aranda BC Reis AR. The impact of maternal consumption of cafeteria diet on reproductive function in the offspring. Physiol Behav (2014) 129:280–6. doi: 10.1016/j.physbeh.2014.03.003 24631302

[33] Wu Y Zhang Z Liao X Wang Z. High fat diet triggers cell cycle arrest and excessive apoptosis of granulosa cells during the follicular development. Biochem Biophys Res Commun (2015) 466(3):599–605. doi: 10.1016/j.bbrc.2015.09.096 26399684

[34] Cheong Y Sadek KH Bruce KD Macklon N Cagampang FR. Diet-induced maternal obesity alters ovarian morphology and gene expression in the adult mouse offspring. Fertil Steril (2014) 102(3):899–907. doi: 10.1016/j.fertnstert.2014.06.015 25063726

[35] Achenbach P Bonifacio E Koczwara K Ziegler A. Natural history of type 1 diabetes. Diabetes (2005) 54(Suppl 2):S25–31. doi: 10.2337/diabetes.54.suppl_2.S25 16306336

[36] Atkinson MA Eisenbarth GS. Type 1 diabetes: new perspectives on disease pathogenesis and treatment. Lancet (9277) 2001:221–9:358. doi: 10.1016/S0140-6736(01)05415-0 11476858

[37] Chakrabarti R Rajagopalan R. Diabetes and insulin resistance associated disorders: disease and the therapy. Curr Sci (2002) 83(12):1533–8.

[38] Dinulovic D Radonjic G. Diabetes mellitus/male infertility. Arch andrology (1990) 25(3):277–93. doi: 10.3109/01485019008987617 2285351

[39] Kahn BB Flier JS. Obesity and insulin resistance. J Clin Invest (2000) 106(4):473–81. doi: 10.1172/JCI10842 PMC380258 10953022

[40] Zatterale F Longo M Naderi J Raciti GA Desiderio A Miele C. Chronic adipose tissue inflammation linking obesity to insulin resistance and type 2 diabetes. Front Physiol (2020) 10. doi: 10.3389/fphys.2019.01607 PMC7000657 32063863

[41] Jensen MD. Adipose tissue as an endocrine organ: implications of its distribution on free fatty acid metabolism. Eur Heart J Suppl (2006) 8(suppl_B):B13–B9. doi: 10.1093/eurheartj/sul003

[42] Amidu N Owiredu WK Alidu H Sarpong C Gyasi-Sarpong CK Quaye L. Association between metabolic syndrome and sexual dysfunction among men with clinically diagnosed diabetes. Diabetol Metab Syndrome (2013) 5(1):1–8. doi: 10.1186/1758-5996-5-42 PMC3733639 23895401

[43] Schoeller EL Schon S Moley KH. The effects of type 1 diabetes on the hypothalamic, pituitary and testes axis. Cell Tissue Res (2012) 349(3):839–47. doi: 10.1007/s00441-012-1387-7 PMC5402694 22526620

[44] Shaikh H Shrivastava VK Amir M. Diabetes mellitus and impairment of male reproductive function: role of hypothalamus pituitary testicular axis and reactive oxygen species. Iranian J Diabetes Obes (2016) 8(1):41–50.

[45] Oghbaei H Hamidian G Alipour MR Alipour S Keyhanmanesh R. The effect of prolonged dietary sodium nitrate treatment on the hypothalamus-pituitary-gonadal axis and testicular structure and function in streptozotocin-induced diabetic male rats. Food Funct (2020) 11(3):2451–65. doi: 10.1039/C9FO00974D 32129362

[46] Livshits A Seidman DS. Fertility issues in women with diabetes. Women’s Health (2009) 5(6):701–7. doi: 10.2217/WHE.09.47 19863473

[47] Oskuye ZZ Bavil FM Hamidian GR Mehri K Qadiri A Ahmadi M. Troxerutin affects the male fertility in prepubertal type 1 diabetic male rats. Iranian J Basic Med Sci (2019) 22(2):197. doi: 10.22038/ijbms.2018.32678.7814 PMC6396992 30834086

[48] La Vignera S Condorelli R Vicari E D’Agata R Calogero AE. Diabetes mellitus and sperm parameters. J Androl (2012) 33(2):145–53. doi: 10.2164/jandrol.111.013193 21474785

[49] Ballester J Muñoz MC Domínguez J Rigau T Guinovart JJ Rodríguez-Gil JE. Insulin-dependent diabetes affects testicular function by FSH-and LH-linked mechanisms. J Androl (2004) 25(5):706–19. doi: 10.1002/j.1939-4640.2004.tb02845.x 15292100

[50] Qadiri A Bavil FM Hamidian G Oskuye ZZ Ahmadi M Oghbaei H. Administration of troxerutin improves testicular function and structure in type-1 diabetic adult rats by reduction of apoptosis. Avicenna J Phytomed (2019) 9(4):374. 31309075 PMC6612250

[51] Dorman JS Steenkiste AR Foley TP Strotmeyer ES Burke JP Kuller LH. Menopause in type 1 diabetic women: is it premature? Diabetes (2001) 50(8):1857–62. doi: 10.2337/diabetes.50.8.1857 11473049

[52] Saei Ghare Naz M Rostami Dovom M Ramezani Tehrani F. The menstrual disturbances in endocrine disorders: a narrative review. Int J Endocrinol Metab (2020) 18(4):e106694. doi: 10.5812/ijem.106694 33613678 PMC7887462

[53] Corleto R Greco C Cacciani M Spaggiari G Simoni M Santi D. Menstrual cycle abnormalities as distinctive sign of type 1 diabetes mellitus: results from a meta-analysis. Endocrine Abstracts (2022). doi: 10.1530/endoabs.81.P83

[54] Lee J Lee HC Kim S-Y Cho GJ Woodruff TK. Poorly-controlled type 1 diabetes mellitus impairs LH-LHCGR signaling in the ovaries and decreases female fertility in mice. Yonsei Med J (2019) 60(7):667–78. doi: 10.3349/ymj.2019.60.7.667 PMC6597468 31250581

[55] Yen I-W Lee C-N Lin M-W Fan K-C Wei J-N Chen K-Y. Overweight and obesity are associated with clustering of metabolic risk factors in early pregnancy and the risk of GDM. PloS One (2019) 14(12):e0225978. doi: 10.1371/journal.pone.0225978 31794594 PMC6890240

[56] Moore TR. Fetal exposure to gestational diabetes contributes to subsequent adult metabolic syndrome. Am J Obstet Gynecol (2010) 202(6):643–9. doi: 10.1016/j.ajog.2010.02.059 20430355

[57] Buchanan TA Xiang AH Page KA. Gestational diabetes mellitus: risks and management during and after pregnancy. Nat Rev Endocrinology (2012) 8(11):639. doi: 10.1038/nrendo.2012.96 22751341 PMC4404707

[58] Brawerman GM Dolinsky VW. Therapies for gestational diabetes and their implications for maternal and offspring health: evidence from human and animal studies. Pharmacol Res (2018) 130:52–73. doi: 10.1016/j.phrs.2018.02.002 29421161

[59] Amiri FN Faramarzi M Bakhtiari A Omidvar S. Risk factors for gestational diabetes mellitus: a case-control study. Am J lifestyle Med (2021) 15(2):184–90. doi: 10.1177/1559827618791980 PMC7958210 33786034

[60] Kim C Newton KM Knopp RH. Gestational diabetes and the incidence of type 2 diabetes: a systematic review. Diabetes Care (2002) 25(10):1862–8. doi: 10.2337/diacare.25.10.1862 12351492

[61] Nankervis A Price S Conn J. Gestational diabetes mellitus: a pragmatic approach to diagnosis and management. Aust J Gen practice (2018) 47(7):445–9. doi: 10.31128/AJGP-01-18-4479 30114871

[62] Bao W Baecker A Song Y Kiely M Liu S Zhang C. Adipokine levels during the first or early second trimester of pregnancy and subsequent risk of gestational diabetes mellitus: a systematic review. Metabolism (2015) 64(6):756–64. doi: 10.1016/j.metabol.2015.01.013 PMC4625979 25749468

[63] Türk G Rişvanlı A Çeribaşı A Sönmez M Yüce A Güvenç M. Effect of gestational diabetes mellitus on testis and pancreatic tissues of male offspring. Andrologia (2018) 50(4):e12976. doi: 10.1111/and.12976 29411891

[64] Mo J-Y Yan Y-S Lin Z-L Liu R Liu X-Q Wu H-Y. Gestational diabetes mellitus suppresses fetal testis development in mice. Biol Reproduction (2022) 107(1):148–56. doi: 10.1093/biolre/ioac138 35774031

[65] Jelodar G Khaksar Z Pourahmadi M. Endocrine profile and testicular histomorphometry in adult rat offspring of diabetic mothers. J Physiol Sci (2009) 59(5):377–82. doi: 10.1007/s12576-009-0045-7 PMC10717756 19536612

[66] Nazari Z Ghafari S Golalipour MJ. Gestational diabetes alters the expression of genes involved in sertoli cells maturation in testis tissue from adult rat offspring. J Anatomical Soc India (2019) 68(2):119. doi: 10.4103/JASI.JASI_22_19

[67] Yang P Read C Kuc RE Buonincontri G Southwood M Torella R. Elabela/Toddler is an endogenous agonist of the apelin APJ receptor in the adult cardiovascular system, and exogenous administration of the peptide compensates for the downregulation of its expression in pulmonary arterial hypertension. Circulation (2017) 135(12):1160–73. doi: 10.1161/CIRCULATIONAHA.116.023218 PMC5363837 28137936

[68] Yamaleyeva LM Shaltout HA Varagic J. Apelin-13 in blood pressure regulation and cardiovascular disease. Curr Opin Nephrol Hypertens (2016) 25(5):396–403. doi: 10.1097/MNH.0000000000000241 27258138

[69] Bertrand C Valet P Castan-Laurell I. Apelin and energy metabolism. Front Physiol (2015) 6:115. doi: 10.3389/fphys.2015.00115 25914650 PMC4392293

[70] Azizi M Iturrioz X Blanchard A Peyrard S De Mota N Chartrel N. Reciprocal regulation of plasma apelin and vasopressin by osmotic stimuli. J Am Soc Nephrology (2008) 19(5):1015–24. doi: 10.1681/ASN.2007070816 PMC2386722 18272843

[71] De Mota N Reaux-Le Goazigo A El Messari S Chartrel N Roesch D Dujardin C. Apelin, a potent diuretic neuropeptide counteracting vasopressin actions through inhibition of vasopressin neuron activity and vasopressin release. Proc Natl Acad Sci (2004) 101(28):10464–9. doi: 10.1073/pnas.0403518101 PMC478592 15231996

[72] O’Carroll A-M Lolait SJ Harris LE Pope GR. The apelin receptor APJ: journey from an orphan to a multifaceted regulator of homeostasis. J Endocrinology (2013) 219(1):R13–35. doi: 10.1530/JOE-13-0227 23943882

[73] O’Carroll A-M Selby TL Palkovits M Lolait SJ. Distribution of mRNA encoding B78/apj, the rat homologue of the human APJ receptor, and its endogenous ligand apelin in brain and peripheral tissues. Biochim Biophys Acta (BBA)-Gene Structure Expression (2000) 1492(1):72–80. doi: 10.1016/S0167-4781(00)00072-5 11004481

[74] Tatemoto K Takayama K Zou M-X Kumaki I Zhang W Kumano K. The novel peptide apelin lowers blood pressure via a nitric oxide-dependent mechanism. Regul peptides (2001) 99(2-3):87–92. doi: 10.1016/S0167-0115(01)00236-1 11384769

[75] Falco MD Fedele V Russo T Virgilio F Sciarrillo R Leone S. Distribution of apelin, the endogenous ligand of the APJ receptor, in the lizard podarcis sicula. J Mol Histology (2004) 35(5):521–7. doi: 10.1007/s10735-004-1247-1 15571329

[76] Ivanov MN Stoyanov DS Pavlov SP Tonchev AB. Distribution, function, and expression of the apelinergic system in the healthy and diseased mammalian brain. Genes (2022) 13(11):1–16. doi: 10.3390/genes13112172 PMC9690544 36421846

[77] Shao ZQ Dou SS Zhu JG Wang HQ Wang CM Cheng BH. Apelin-13 inhibits apoptosis and excessive autophagy in cerebral ischemia/reperfusion injury. Neural Regen Res (2021) 16(6):1044–51. doi: 10.4103/1673-5374.300725 PMC8224111 33269749

[78] Chapman FA Nyimanu D Maguire JJ Davenport AP Newby DE Dhaun N. The therapeutic potential of apelin in kidney disease. Nat Rev Nephrology (2021) 17(12):840–53. doi: 10.1038/s41581-021-00461-z PMC8361827 34389827

[79] Chen J Chen X Li S Jiang Y Mao H Zhang R. Individual phosphorylation sites at the c-terminus of the apelin receptor play different roles in signal transduction. Redox Biol (2020) 36:101629. doi: 10.1016/j.redox.2020.101629 32863206 PMC7338617

[80] Estienne A Bongrani A Froment P Dupont J. Apelin and chemerin receptors are G protein-coupled receptors involved in metabolic as well as reproductive functions: potential therapeutic implications? Curr Opin Endocrine Metab Res (2021) 16:86–95. doi: 10.1016/j.coemr.2020.09.005

[81] Zeng XJ Yu SP Zhang L Wei L. Neuroprotective effect of the endogenous neural peptide apelin in cultured mouse cortical neurons. Exp Cell Res (2010) 316(11):1773–83. doi: 10.1016/j.yexcr.2010.02.005 PMC3155990 20152832

[82] Xin Q Cheng B Pan Y Liu H Chen J Bai B. Neuroprotective effects of apelin-13 on experimental ischemic stroke through suppression of inflammation. Peptides (2015) 63:55–62. doi: 10.1016/j.peptides.2014.09.016 25278489

[83] Reaux-Le Goazigo A Alvear-Perez R Zizzari P Epelbaum J Bluet-Pajot M-T Llorens-Cortes C. Cellular localization of apelin and its receptor in the anterior pituitary: evidence for a direct stimulatory action of apelin on ACTH release. Am J Physiol Endocrinol Metab (2007) 292(1):E7–E15. doi: 10.1152/ajpendo.00521.2005 16896162

[84] Antushevich H Wójcik M. Apelin in disease. Clinica chimica Acta (2018) 483:241–8. doi: 10.1016/j.cca.2018.05.012 29750964

[85] Taheri S Murphy K Cohen M Sujkovic E Kennedy A Dhillo W. The effects of centrally administered apelin-13 on food intake, water intake and pituitary hormone release in rats. Biochem Biophys Res Commun (2002) 291(5):1208–12. doi: 10.1006/bbrc.2002.6575 11883945

[86] Reaux-Le Goazigo A Bodineau L De Mota N Jeandel L Chartrel N Knauf C. Apelin and the proopiomelanocortin system: a new regulatory pathway of hypothalamic α-MSH release. Am J Physiol Endocrinol Metab (2011) 301(5):E955–E66. doi: 10.1152/ajpendo.00090.2011 21846903

[87] Reaux A De Mota N Skultetyova I Lenkei Z El Messari S Gallatz K. Physiological role of a novel neuropeptide, apelin, and its receptor in the rat brain. J Neurochem (2001) 77(4):1085–96. doi: 10.1046/j.1471-4159.2001.00320.x 11359874

[88] Goazigo AR-L Morinville A Burlet A Llorens-Cortes C Beaudet A. Dehydration-induced cross-regulation of apelin and vasopressin immunoreactivity levels in magnocellular hypothalamic neurons. Endocrinology (2004) 145(9):4392–400. doi: 10.1210/en.2004-0384 15166125

[89] Hus-Citharel A Bodineau L Frugière A Joubert F Bouby N Llorens-Cortes C. Apelin counteracts vasopressin-induced water reabsorption *via* cross talk between apelin and vasopressin receptor signaling pathways in the rat collecting duct. Endocrinology (2014) 155(11):4483–93. doi: 10.1210/en.2014-1257 25157454

[90] Boulkeroua C Ayari H Khalfaoui T Lafrance M Besserer-Offroy É Ekindi N. Apelin-13 regulates vasopressin-induced aquaporin-2 expression and trafficking in kidney collecting duct cells. Cell Physiol Biochem (2019) 53(4):687–700. doi: 10.33594/000000165 31577078

[91] Roberts EM Newson MJ Pope GR Landgraf R Lolait SJ O’Carroll A-M. Abnormal fluid homeostasis in apelin receptor knockout mice. J Endocrinol (2009) 202(3):453. doi: 10.1677/JOE-09-0134 19578099 PMC2729781

[92] Kuba K Zhang L Imai Y Arab S Chen M Maekawa Y. Impaired heart contractility in apelin gene–deficient mice associated with aging and pressure overload. Circ Res (2007) 101(4):e32–42. doi: 10.1161/CIRCRESAHA.107.158659 17673668

[93] Kawamata Y Habata Y Fukusumi S Hosoya M Fujii R Hinuma S. Molecular properties of apelin: tissue distribution and receptor binding. Biochim Biophys Acta (BBA)-Molecular Cell Res (2001) 1538(2-3):162–71. doi: 10.1016/S0167-4889(00)00143-9 11336787

[94] Clarke KJ Whitaker KW Reyes TM. Diminished metabolic responses to centrally-administered apelin-13 in diet-induced obese rats fed a high-fat diet. J Neuroendocrinology (2009) 21(2):83–9. doi: 10.1111/j.1365-2826.2008.01815.x 19076266

[95] Mitra A Katovich MJ Mecca A Rowland NE. Effects of central and peripheral injections of apelin on fluid intake and cardiovascular parameters in rats. Physiol Behav (2006) 89(2):221–5. doi: 10.1016/j.physbeh.2006.06.006 16839572

[96] Flahault A Girault-Sotias P-E Keck M Alvear-Perez R De Mota N Estéoulle L. A metabolically stable apelin-17 analog decreases AVP-induced antidiuresis and improves hyponatremia. Nat Commun (2021) 12(1):305. doi: 10.1038/s41467-020-20560-y 33436646 PMC7804859

[97] Sato K Takahashi T Kobayashi Y Hagino A Roh SG Katoh K. Apelin is involved in postprandial responses and stimulates secretion of arginine-vasopressin, adrenocorticotropic hormone, and growth hormone in the ruminant. Domest Anim Endocrinology (2012) 42(3):165–72. doi: 10.1016/j.domaniend.2011.11.006 22177697

[98] Papadopoulos DP Makris T Perrea D Zerva K Tsioufis C Faselis C. Apelin and relaxin plasma levels in young healthy offspring of patients with essential hypertension. J Clin Hypertension (2014) 16(3):198–201. doi: 10.1111/jch.12260 PMC8032001 24708381

[99] Than A Zhang X Leow MK Poh CL Chong SK Chen P. Apelin attenuates oxidative stress in human adipocytes. J Biol Chem (2014) 289(6):3763–74. doi: 10.1074/jbc.M113.526210 PMC3916573 24362107

[100] Chun HJ Ali ZA Kojima Y Kundu RK Sheikh AY Agrawal R. Apelin signaling antagonizes ang II effects in mouse models of atherosclerosis. J Clin Invest (2008) 118(10):3343–54. doi: 10.1172/JCI34871 PMC2525695 18769630

[101] Du J-H Li X Li R Xu L Ma R-R Liu S-F. Elevation of serum apelin-13 associated with proliferative diabetic retinopathy in type 2 diabetic patients. Int J Ophthal (2014) 7(6):968. doi: 10.3980/j.issn.2222-3959.2014.06.10 25540748 PMC4270990

[102] Szokodi I Tavi P Foüldes GB Voutilainen-Myllylaü S Ilves M Tokola H. Apelin, the novel endogenous ligand of the orphan receptor APJ, regulates cardiac contractility. Circ Res (2002) 91(5):434–40. doi: 10.1161/01.RES.0000033522.37861.69 12215493

[103] Wang C Du J-F Wu F Wang H-C. Apelin decreases the SR Ca2+ content but enhances the amplitude of [Ca2+] i transient and contractions during twitches in isolated rat cardiac myocytes. Am J Physiology-Heart Circulatory Physiol (2008) 294(6):H2540–H6. doi: 10.1152/ajpheart.00046.2008 18424641

[104] Dray C Knauf C Daviaud D Waget A Boucher J Buléon M. Apelin stimulates glucose utilization in normal and obese insulin-resistant mice. Cell Metab (2008) 8(5):437–45. doi: 10.1016/j.cmet.2008.10.003 19046574

[105] Yue P Jin H Aillaud M Deng AC Azuma J Asagami T. Apelin is necessary for the maintenance of insulin sensitivity. Am J physiology-endocrinology Metab (2010) 298(1):E59–67. doi: 10.1152/ajpendo.00385.2009 PMC2806109 19861585

[106] Hu G Wang Z Zhang R Sun W Chen X. The role of apelin/apelin receptor in energy metabolism and water homeostasis: a comprehensive narrative review. Front Physiol (2021) 12:632886. doi: 10.3389/fphys.2021.632886 33679444 PMC7928310

[107] Winzell MS Magnusson C Ahrén B. The apj receptor is expressed in pancreatic islets and its ligand, apelin, inhibits insulin secretion in mice. Regul peptides (2005) 131(1-3):12–7. doi: 10.1016/j.regpep.2005.05.004 15970338

[108] Ringström C Nitert MD Bennet H Fex M Valet P Rehfeld JF. Apelin is a novel islet peptide. Regul Peptides (2010) 162(1):44–51. doi: 10.1016/j.regpep.2010.03.005 20346374

[109] Guo L Li Q Wang W Yu P Pan H Li P. Apelin inhibits insulin secretion in pancreatic β-cells by activation of PI3-kinase-phosphodiesterase 3B. Endocrine Res (2009) 34(4):142–54. doi: 10.3109/07435800903287079 19878074

[110] Coughlan KA Valentine RJ Ruderman NB Saha AK. AMPK activation: a therapeutic target for type 2 diabetes? Diabetes Metab syndrome obesity: Targets Ther (2014) 7:241. doi: 10.2147/DMSO.S43731 PMC4075959 25018645

[111] Castan-Laurell I Dray C Attané C Duparc T Knauf C Valet P. Apelin, diabetes, and obesity. Endocrine (2011) 40(1):1–9. doi: 10.1007/s12020-011-9507-9 21725702

[112] Zhu S Sun F Li W Cao Y Wang C Wang Y. Apelin stimulates glucose uptake through the PI3K/Akt pathway and improves insulin resistance in 3T3-L1 adipocytes. Mol Cell Biochem (2011) 353(1):305–13. doi: 10.1007/s11010-011-0799-0 21461612

[113] Xu S Han P Huang M Wu JC Chang C Tsao PS. *In vivo*, ex vivo, and *in vitro* studies on apelin’s effect on myocardial glucose uptake. Peptides (2012) 37(2):320–6. doi: 10.1016/j.peptides.2012.08.004 22906703

[114] Attané C Foussal C Le Gonidec S Benani A Daviaud D Wanecq E. Apelin treatment increases complete fatty acid oxidation, mitochondrial oxidative capacity, and biogenesis in muscle of insulin-resistant mice. Diabetes (2012) 61(2):310–20. doi: 10.2337/db11-0100 PMC3266414 22210322

[115] Li C Cheng H Adhikari BK Wang S Yang N Liu W. The role of apelin-APJ system in diabetes and obesity. Front endocrinology (2022) 13:820002. doi: 10.3389/fendo.2022.820002 PMC8959308 35355561

[116] Dray C Sakar Y Vinel C Daviaud D Masri B Garrigues L. The intestinal glucose–apelin cycle controls carbohydrate absorption in mice. Gastroenterology (2013) 144(4):771–80. doi: 10.1053/j.gastro.2013.01.004 23313268

[117] Fukaya M Mizuno A Arai H Muto K Uebanso T Matsuo K. Mechanism of rapid-phase insulin response to elevation of portal glucose concentration. Am J Physiology-Endocrinology Metab (2007) 293(2):E515–E22. doi: 10.1152/ajpendo.00536.2006 17473051

[118] Yamagata K Tagawa C Matsufuji H Chino M. Dietary apigenin regulates high glucose and hypoxic reoxygenation-induced reductions in apelin expression in human endothelial cells. J Nutr Biochem (2012) 23(8):929–36. doi: 10.1016/j.jnutbio.2011.04.019 21852087

[119] Duparc T Colom A Cani PD Massaly N Rastrelli S Drougard A. Central apelin controls glucose homeostasis *via* a nitric oxide-dependent pathway in mice. Antioxidants Redox Signaling (2011) 15(6):1477–96. doi: 10.1089/ars.2010.3454 21395477

[120] Drougard A Duparc T Brenachot X Carneiro L Gouazé A Fournel A. Hypothalamic apelin/reactive oxygen species signaling controls hepatic glucose metabolism in the onset of diabetes. Antioxidants Redox Signaling (2014) 20(4):557–73. doi: 10.1089/ars.2013.5182 PMC3901354 23879244

[121] Yue P Jin H Xu S Aillaud M Deng AC Azuma J. Apelin decreases lipolysis *via* gq, gi, and AMPK-dependent mechanisms. Endocrinology (2011) 152(1):59–68. doi: 10.1210/en.2010-0576 21047945 PMC3033059

[122] Than A Cheng Y Foh L-C Leow MK-S Lim SC Chuah YJ. Apelin inhibits adipogenesis and lipolysis through distinct molecular pathways. Mol Cell Endocrinol (2012) 362(1-2):227–41. doi: 10.1016/j.mce.2012.07.002 22842084

[123] Mehri K Babri S. The effect of troxerutin on apelin-13 and its receptor gene expression in ovarian of pregnant rats fed a high-fat diet. J Advan Biomed Sci (2022) :12(3):3958–66. doi: 10.18502/jabs.v11i3.8789

[124] Yang H Zhao L Zhang J Tang CS Qi YF Zhang J. Effect of treadmill running on apelin and APJ expression in adipose tissue and skeletal muscle in rats fed a high-fat diet. Int J Sports Med (2015) 36(7):535–41. doi: 10.1055/s-0034-1398653 25781869

[125] Garcia-Diaz DF Campion J Arellano AV Milagro FI Moreno-Aliaga MJ Martinez JA. Fat intake leads to differential response of rat adipocytes to glucose, insulin and ascorbic acid. Exp Biol Med (Maywood) (2012) 237(4):407–16. doi: 10.1258/ebm.2011.011317 22454546

[126] Marousez L Hanssens S Butruille L Petit C Pourpe C Besengez C. Breast milk apelin level increases with maternal obesity and high-fat feeding during lactation. Int J Obes (Lond) (2021) 45(5):1052–60. doi: 10.1038/s41366-021-00772-y 33594258

[127] Masuyama H Mitsui T Eguchi T Tamada S Hiramatsu Y. The effects of paternal high-fat diet exposure on offspring metabolism with epigenetic changes in the mouse adiponectin and leptin gene promoters. Am J Physiology-Endocrinology Metab (2016) 311(1):E236–E45. doi: 10.1152/ajpendo.00095.2016 27245335

[128] Alipour FG Ashoori MR Pilehvar-Soltanahmadi Y Zarghami N. An overview on biological functions and emerging therapeutic roles of apelin in diabetes mellitus. Diabetes Metab Syndr (2017) 11 Suppl 2:S919–s23. doi: 10.1016/j.dsx.2017.07.016 28712823

[129] Hoseindoost M Alipour MR Farajdokht F Diba R Bayandor P Mehri K. Effects of troxerutin on inflammatory cytokines and BDNF levels in male offspring of high-fat diet fed rats. Avicenna J Phytomed (2019) 9(6):597–605. doi: 10.22038/AJP.2019.13587 31763218 PMC6823526

[130] Soriguer F Garrido-Sanchez L Garcia-Serrano S Garcia-Almeida JM Garcia-Arnes J Tinahones FJ. Apelin levels are increased in morbidly obese subjects with type 2 diabetes mellitus. Obes surgery (2009) 19(11):1574–80. doi: 10.1007/s11695-009-9955-y 19756893

[131] Habchi M Duvillard L Cottet V Brindisi MC Bouillet B Beacco M. Circulating a pelin is increased in patients with type 1 or type 2 diabetes and is associated with better glycaemic control. Clin endocrinology (2014) 81(5):696–701. doi: 10.1111/cen.12404 24417455

[132] Castan-Laurell I Vítkova M Daviaud D Dray C Kováčiková M Kovacova Z. Effect of hypocaloric diet-induced weight loss in obese women on plasma apelin and adipose tissue expression of apelin and APJ. Eur J endocrinology (2008) 158(6):905–10. doi: 10.1530/EJE-08-0039 PMC2683032 18390990

[133] Grønning LM Cederberg A Miura N Enerbaück S Taskeín K. Insulin and TNFα induce expression of the forkhead transcription factor gene Foxc2 in 3T3-L1 adipocytes *via* PI3K and ERK 1/2-dependent pathways. Mol Endocrinology (2002) 16(4):873–83. doi: 10.1210/mend.16.4.0803 11923482

[134] Kabaran S Besler HT. Do fatty acids affect fetal programming? J Health Population Nutr (2015) 33(1):1–9. doi: 10.1186/s41043-015-0018-9 PMC5025983 26825664

[135] Yamamoto T Habata Y Matsumoto Y Yasuhara Y Hashimoto T Hamajyo H. Apelin-transgenic mice exhibit a resistance against diet-induced obesity by increasing vascular mass and mitochondrial biogenesis in skeletal muscle. Biochim Biophys Acta (BBA)-General Subjects (2011) 1810(9):853–62. doi: 10.1016/j.bbagen.2011.05.004 21609753

[136] Wang ZV Scherer PE. Adiponectin, the past two decades. J Mol Cell Biol (2016) 8(2):93–100. doi: 10.1093/jmcb/mjw011 26993047 PMC4816148

[137] Ryo M Nakamura T Kihara S Kumada M Shibazaki S Takahashi M. Adiponectin as a biomarker of the metabolic syndrome. Circ J (2004) 68(11):975–81. doi: 10.1253/circj.68.975 15502375

[138] Frühbeck G Catalán V Rodríguez A Ramírez B Becerril S Salvador J. Adiponectin-leptin ratio is a functional biomarker of adipose tissue inflammation. Nutrients (2019) 11(2)::1–13. doi: 10.3390/nu11020454 PMC6412349 30813240

[139] Higuchi K Masaki T Gotoh K Chiba S Katsuragi I Tanaka K. Apelin, an APJ receptor ligand, regulates body adiposity and favors the messenger ribonucleic acid expression of uncoupling proteins in mice. Endocrinology (2007) 148(6):2690–7. doi: 10.1210/en.2006-1270 17347313

[140] Attané C Foussal C Le Gonidec S Benani A Daviaud D Wanecq E. Apelin treatment increases complete fatty acid oxidation, mitochondrial oxidative capacity, and biogenesis in muscle of insulin-resistant mice. Diabetes (2012) 61(2):310–20. doi: 10.2337/db11-0100 PMC3266414 22210322

[141] Alipour FG Ashoori MR Pilehvar-Soltanahmadi Y Zarghami N. An overview on biological functions and emerging therapeutic roles of apelin in diabetes mellitus. Diabetes Metab Syndrome: Clin Res Rev (2017) 11:S919–S23. doi: 10.1016/j.dsx.2017.07.016 28712823

[142] Li C Cheng H Adhikari BK Wang S Yang N Liu W. The role of apelin–APJ system in diabetes and obesity. Front Endocrinol (2022) 13. doi: 10.3389/fendo.2022.820002 PMC8959308 35355561

[143] Feng J Zhao H Du M Wu X. The effect of apelin-13 on pancreatic islet beta cell mass and myocardial fatty acid and glucose metabolism of experimental type 2 diabetic rats. Peptides (2019) 114:1–7. doi: 10.1016/j.peptides.2019.03.006 30954534

[144] Xu S Philip S Tsao PY. Apelin insulin resistance: another arrow for the quiver? J Diabetes (2011) 3(3):225–31. doi: 10.1111/j.1753-0407.2011.00132.x PMC3156858 21631898

[145] Dray C Knauf C Daviaud D Waget A Boucher J Buléon M. Apelin stimulates glucose utilization in normal and obese insulin-resistant mice. Cell Metab (2008) 8(5):437–45. doi: 10.1016/j.cmet.2008.10.003 19046574

[146] Chen H Zheng C Zhang X Li J Li J Zheng L. Apelin alleviates diabetes-associated endoplasmic reticulum stress in the pancreas of akita mice. Peptides (2011) 32(8):1634–9. doi: 10.1016/j.peptides.2011.06.025 21762740

[147] Li M Fang H Hu J. Apelin−13 ameliorates metabolic and cardiovascular disorders in a rat model of type 2 diabetes with a high−fat diet. Mol Med Rep (2018) 18(6):5784–90. doi: 10.3892/mmr.2018.9607 30387843

[148] Seshadri R. American Diabetes association gestational diabetes mellitus. Diabetes Care (2002) 25:S94–S6. doi: 10.2337/diacare.25.2007.S94

[149] Mayeur S Wattez J-S Lukaszewski M-A Lecoutre S Butruille L Drougard A. Apelin controls fetal and neonatal glucose homeostasis and is altered by maternal undernutrition. Diabetes (2016) 65(3):554–60. doi: 10.2337/db15-0228 26631739

[150] Sun J Ren J Zuo C Deng D Pan F Chen R. Circulating apelin, chemerin and omentin levels in patients with gestational diabetes mellitus: a systematic review and meta-analysis. Lipids Health disease (2020) 19(1):1–15. doi: 10.1186/s12944-020-01209-7 PMC7035755 32087711

[151] De Gennaro G Palla G Battini L Simoncini T Del Prato S Bertolotto A. The role of adipokines in the pathogenesis of gestational diabetes mellitus. Gynecological Endocrinol (2019) 35(9):737–51. doi: 10.1080/09513590.2019.1597346 30990092

[152] Boyadzhieva M Atanasova I Zacharieva S Kedikova S. Adipocytokines during pregnancy and postpartum in women with gestational diabetes and healthy controls. J endocrinological Invest (2013) 36(11):944–9. doi: 10.3275/8968 23685996

[153] Kourtis A Gkiomisi A Mouzaki M Makedou K Anastasilakis AD Toulis KA. Apelin levels in normal pregnancy. Clin endocrinology (2011) 75(3):367–71. doi: 10.1111/j.1365-2265.2011.04061.x 21521329

[154] Sun J Ren J Zuo C Deng D Pan F Chen R. Circulating apelin, chemerin and omentin levels in patients with gestational diabetes mellitus: a systematic review and meta-analysis. Lipids Health Disease (2020) 19(1):26. doi: 10.1186/s12944-020-01209-7 PMC7035755 32087711

[155] Aslan M Celik O Celik N Turkcuoglu I Yilmaz E Karaer A. Cord blood nesfatin-1 and apelin-36 levels in gestational diabetes mellitus. Endocrine (2012) 41(3):424–9. doi: 10.1007/s12020-011-9577-8 22203468

[156] Pérez-López FR Wu J-N Yao L López-Baena MT Pérez-Roncero GR Varikasuvu SR. Apelin levels in pregnant women with and without gestational diabetes mellitus: a collaborative systematic review and meta-analysis. Gynecological Endocrinology (2022) 38(10):803–12. doi: 10.1080/09513590.2022.2114450 36002980

[157] Boucher JRM Masri B Daviaud DL Gesta SP Guigneí C Mazzucotelli A. Apelin, a newly identified adipokine up-regulated by insulin and obesity. Endocrinology (2005) 146(4):1764–71. doi: 10.1210/en.2004-1427 15677759

[158] Colledge WH Mei H de Tassigny X. Mouse models to study the central regulation of puberty. Mol Cell Endocrinol (2010) 324(1-2):12–20. doi: 10.1016/j.mce.2010.01.015 20083157

[159] Czarzasta K Cudnoch-Jedrzejewska A. The role of the apelinergic and vasopressinergic systems in the regulation of the cardiovascular system and the pathogenesis of cardiovascular disease. Kardiologia Polska (Polish Heart Journal) (2014) 72(2):122–5. doi: 10.5603/KP.2014.0028 24604503

[160] De Mota N Lenkei Z Llorens-Cortès C. Cloning, pharmacological characterization and brain distribution of the rat apelin receptor. Neuroendocrinology (2000) 72(6):400–7. doi: 10.1159/000054609 11146423

[161] O’Carroll AM Lolait SJ. Regulation of rat APJ receptor messenger ribonucleic acid expression in magnocellular neurones of the paraventricular and supraopric nuclei by osmotic stimuli. J Neuroendocrinol (2003) 15(7):661–6. doi: 10.1046/j.1365-2826.2003.01044.x 12787050

[162] Pope GR Roberts EM Lolait SJ O’Carroll A-M. Central and peripheral apelin receptor distribution in the mouse: species differences with rat. Peptides (2012) 33(1):139–48. doi: 10.1016/j.peptides.2011.12.005 PMC3314948 22197493

[163] Reaux A De Mota N Skultetyova I Lenkei Z El Messari S Gallatz K. Physiological role of a novel neuropeptide, apelin, and its receptor in the rat brain. J Neurochem (2001) 77(4):1085–96. doi: 10.1046/j.1471-4159.2001.00320.x 11359874

[164] Shimizu T Kosaka N Murayama C Tetsuka M Miyamoto A. Apelin and APJ receptor expression in granulosa and theca cells during different stages of follicular development in the bovine ovary: involvement of apoptosis and hormonal regulation. Anim Reprod Sci (2009) 116(1-2):28–37. doi: 10.1016/j.anireprosci.2009.01.009 19223129

[165] Tekin S Erden Y Sandal S Etem Onalan E Ozyalin F Ozen H. Effects of apelin on reproductive functions: relationship with feeding behavior and energy metabolism. Arch Physiol Biochem (2017) 123(1):9–15. doi: 10.1080/13813455.2016.1211709 27494693

[166] Şişli HB Hayal TB Şenkal S Kıratlı B Sağraç D Seçkin S. Apelin receptor signaling protects GT1-7 GnRH neurons against oxidative stress in vitro. Cell Mol Neurobiol (2022) 42(3):753–75. doi: 10.1007/s10571-020-00968-2 PMC11441187 32989586

[167] Kacar E Ercan Z Serhatlioglu I Sumer A Kelestimur H Kutlu S. The effects of apelin on myometrium contractions in pregnant rats. Cell Mol Biol (2018) 64(11):74–9. doi: 10.14715/cmb/2018.64.11.13 30213292

[168] Hehir MP Morrison JJ. The adipokine apelin and human uterine contractility. Am J obstetrics gynecology (2012) 206(4):359.e1–.e5. doi: 10.1016/j.ajog.2012.01.032 22360921

[169] Asalah AK Attia KI Mahdi SE-S. Apelin induced modulation of uterine contractility in adult albino rats and its possible mechanism/s of action. Zagazig Univ Med J (2020) 26(1):174–85. doi: 10.21608/zumj.2019.11148.1157

[170] Carvajal JA Oporto JI. The myometrium in pregnant women with obesity. Curr Vasc Pharmacol (2021) 19(2):193–200. doi: 10.2174/1570161118666200525133530 32484103

[171] Schilffarth S Antoni B Schams D Meyer HH Berisha B. The expression of apelin and its receptor APJ during different physiological stages in the bovine ovary. Int J Biol Sci (2009) 5(4):344. doi: 10.7150/ijbs.5.344 19461937 PMC2684680

[172] Shokrollahi B Zheng H-Y Li L-Y Tang L-P Ma X-Y Lu X-R. Apelin and apelin receptor in follicular granulosa cells of buffalo ovaries: expression and regulation of steroidogenesis. Front Endocrinol (2022) 13. doi: 10.3389/fendo.2022.844360 PMC8960050 35355567

[173] Roche J Ramé C Reverchon M Mellouk N Cornuau M Guerif F. Apelin (APLN) and apelin receptor (APLNR) in human ovary: expression, signaling, and regulation of steroidogenesis in primary human luteinized granulosa cells. Biol Reproduction (2016) 95(5):104, 1–12. doi: 10.1095/biolreprod.116.141754 27683264

[174] Rak A Drwal E Rame C Knapczyk-Stwora K Słomczyńska M Dupont J. Expression of apelin and apelin receptor (APJ) in porcine ovarian follicles and *in vitro* effect of apelin on steroidogenesis and proliferation through APJ activation and different signaling pathways. Theriogenology (2017) 96:126–35. doi: 10.1016/j.theriogenology.2017.04.014 28532828

[175] Roche J Ramé C Reverchon M Mellouk N Rak A Froment P. Apelin (APLN) regulates progesterone secretion and oocyte maturation in bovine ovarian cells. Reproduction (2017) 153(5):589–603. doi: 10.1530/REP-16-0677 28250234

[176] Gupta M Korde JP Bahiram KB Sardar VM Kurkure NV. Expression and localization of apelin and apelin receptor (APJ) in buffalo ovarian follicles and corpus luteum and the *in-vitro* effect of apelin on steroidogenesis and survival of granulosa cells. Theriogenology (2023) 197:240–51. doi: 10.1016/j.theriogenology.2022.12.013 36525863

[177] Shuang L Jidong W Hongjuan P Zhenwei Y. Effects of apelin on proliferation and apoptosis in rat ovarian granulosa cells. Clin Exp Obstetrics Gynecology (2016) 43(3):409–13. doi: 10.12891/ceog2133.2016 27328502

[178] Shokrollahi B Zheng H-Y Ma X-Y Shang J-H. The effects of apelin on IGF1/FSH-induced steroidogenesis, proliferation, bax expression, and total antioxidant capacity in granulosa cells of buffalo ovarian follicles. Veterinary Res Commun (2023). doi: 10.1007/s11259-023-10107-z 37036601

[179] Shirasuna K Shimizu T Sayama K Asahi T Sasaki M Berisha B. Expression and localization of apelin and its receptor APJ in the bovine corpus luteum during the estrous cycle and prostaglandin F2a-induced luteolysis. Reproduction (2008) 135(4):519–26. doi: 10.1530/REP-07-0409 18367512

[180] Kasai A Shintani N Oda M Kakuda M Hashimoto H Matsuda T. Apelin is a novel angiogenic factor in retinal endothelial cells. Biochem Biophys Res Commun (2004) 325(2):395–400. doi: 10.1016/j.bbrc.2004.10.042 15530405

[181] Dogra S Neelakantan D Patel MM Griesel B Olson A Woo S. Adipokine Apelin/APJ pathway promotes peritoneal dissemination of ovarian cancer cells by regulating lipid MetabolismApelin/APJ regulates ovarian cancer lipid metabolism. Mol Cancer Res (2021) 19(9):1534–45. doi: 10.1158/1541-7786.MCR-20-0991 PMC11486291 34172534

[182] Liu Q Jiang J Shi Y Mo Z Li M. Apelin/Apelin receptor: a new therapeutic target in polycystic ovary syndrome. Life Sci (2020) 260:118310. doi: 10.1016/j.lfs.2020.118310 32835696

[183] Hasan MM Abd El Hameed AA. Serum adipokine (apelin) in lean and obese polycystic ovary syndrome patients before and after metformin treatment. Middle East Fertility Soc J (2018) 23(4):315–8. doi: 10.1016/j.mefs.2018.04.003

[184] Sun X Wu X Zhou Y Yu X Zhang W. Evaluation of apelin and insulin resistance in patients with PCOS and therapeutic effect of drospirenone-ethinylestradiol plus metformin. Med Sci monitor: Int Med J Exp Clin Res (2015) 21:2547. doi: 10.12659/MSM.894926 PMC4556163 26314870

[185] Chang C-Y Tsai Y-C Lee C-H Chan T-F Wang S-H Su J-H. Lower serum apelin levels in women with polycystic ovary syndrome. Fertility Sterility (2011) 95(8):2520–3.e2. doi: 10.1016/j.fertnstert.2011.04.044 21575945

[186] Olszanecka-Glinianowicz M Madej P Nylec M Owczarek A Szanecki W Skałba P. Circulating apelin level in relation to nutritional status in polycystic ovary syndrome and its association with metabolic and hormonal disturbances. Clin Endocrinology (2013) 79(2):238–42. doi: 10.1111/cen.12120 23199261

[187] Mishra P Mittal P Rani A Bharti R Agarwal V Suri J. Adiponectin to leptin ratio and its association with insulin resistance in women with polycystic ovarian syndrome. Indian J Endocrinol Metab (2022) 26(3):239–44. doi: 10.4103/ijem.ijem_137_22 PMC9555382 36248039

[188] Neelakantan D Dogra S Devapatla B Jaiprasart P Mukashyaka MC Janknecht R. Multifunctional APJ pathway promotes ovarian cancer progression and MetastasisThe APJ pathway promotes ovarian cancer progression. Mol Cancer Res (2019) 17(6):1378–90. doi: 10.1158/1541-7786.MCR-18-0989 PMC6548659 30858172

[189] Jang IY Lee S Kim JH Lee E Lee JY Park SJ. Lack of association between circulating apelin level and frailty-related functional parameters in older adults: a cross-sectional study. BMC geriatrics (2020) 20(1):420. doi: 10.1186/s12877-020-01837-9 33087053 PMC7579806

[190] Vinel C Lukjanenko L Batut A Deleruyelle S Pradere J-P Le Gonidec S. The exerkine apelin reverses age-associated sarcopenia. Nat Med (2018) 24(9):1360–71. doi: 10.1038/s41591-018-0131-6 30061698

[191] Duman H Bahçeci I Hamur H Demirelli S Ramazan Dilek A Erdogan T. The relationship between serum apelin levels and the severity of calcific aortic stenosis. Acta Cardiologica Sinica (2018) 34(3):259–66. doi: 10.6515/ACS.201805_34(3).20180207A PMC5968342 29844647

[192] Rai R Ghosh AK Eren M Mackie AR Levine DC Kim S-Y. Downregulation of the apelinergic axis accelerates aging, whereas its systemic restoration improves the mammalian healthspan. Cell Rep (2017) 21(6):1471–80. doi: 10.1016/j.celrep.2017.10.057 PMC5892413 29117554

[193] Troisi A Dall’Aglio C Maranesi M Orlandi R Pastore S Bazzano M. Presence and localization of apelin and its cognate receptor in canine testes using immunohistochemical and RT-PCR techniques. Vet Res Commun (2023) 47(2):929–35. doi: 10.21203/rs.3.rs-1266418/v2 PMC10209344 36331787

[194] Brzoskwinia M Pardyak L Rak A Kaminska A Hejmej A Marek S. Flutamide alters the expression of chemerin, apelin, and vaspin and their respective receptors in the testes of adult rats. Int J Mol Sci (2020) 21(12):4439. doi: 10.3390/ijms21124439 32580404 PMC7378763

[195] Song K Yang X An G Xia X Zhao J Xu X. Targeting APLN/APJ restores blood-testis barrier and improves spermatogenesis in murine and human diabetic models. Nat Commun (2022) 13(1):1–17. doi: 10.1038/s41467-022-34990-3 36443325 PMC9705293

[196] Dupont J Pollet-Villard X Reverchon M Mellouk N Levy R. Adipokines in human reproduction. Hormone Mol Biol Clin Invest (2015) 24(1):11–24. doi: 10.1515/hmbci-2015-0034 26574894

[197] Akkan SS İzgüt-Uysal VN Çakır T Özbey Ö Üstünel İ. The effect of experimental varicocele on the apelin and APJ expressions in rat testis tissue. Tissue Cell (2020) 63:101318. doi: 10.1016/j.tice.2019.101318 32223946

[198] Das M Annie L Derkach KV Shpakov AO Gurusubramanian G Roy VK. Expression and localization of apelin and its receptor in the testes of diabetic mice and its possible role in steroidogenesis. Cytokine (2021) 144:155554. doi: 10.1016/j.cyto.2021.155554 33962842

[199] Song K Yang X An G Xia X Zhao J Xu X. Targeting APLN/APJ restores blood-testis barrier and improves spermatogenesis in murine and human diabetic models. Nat Commun (2022) 13(1):7335. doi: 10.1038/s41467-022-34990-3 36443325 PMC9705293

